# Targeting Glycolysis in Alloreactive T Cells to Prevent Acute Graft-*Versus*-Host Disease While Preserving Graft-Versus-Leukemia Effect

**DOI:** 10.3389/fimmu.2022.751296

**Published:** 2022-02-28

**Authors:** Ying Huang, Yujing Zou, Yiqun Jiao, Peijie Shi, Xiaoli Nie, Wei Huang, Chuanfeng Xiong, Michael Choi, Charles Huang, Andrew N. Macintyre, Amanda Nichols, Fang Li, Chuan-Yuan Li, Nancie J. MacIver, Diana M. Cardona, Todd V. Brennan, Zhiguo Li, Nelson J. Chao, Jeffrey C. Rathmell, Benny J. Chen

**Affiliations:** ^1^Division of Hematologic Malignancies and Cellular Therapy/Bone Marrow Transplantation (BMT), Department of Medicine, Duke University Medical Center, Durham, NC, United States; ^2^Departments of Pharmacology and Cancer Biology, Duke University Medical Center, Durham, NC, United States; ^3^Department of Pediatrics, Duke University Medical Center, Durham, NC, United States; ^4^Department of Dermatology, Duke University Medical Center, Durham, NC, United States; ^5^Duke Cancer Institute, Duke University Medical Center, Durham, NC, United States; ^6^Department of Immunology, Duke University Medical Center, Durham, NC, United States; ^7^Department of Pathology, Duke University Medical Center, Durham, NC, United States; ^8^Department of Surgery, Cedars-Sinai Medical Center, Los Angeles, CA, United States; ^9^Department of Biostatistics and Bioinformatics, Duke University Medical Center, Durham, NC, United States; ^10^Vanderbilt Center for Immunobiology, Departments of Pathology, Microbiology, and Immunology, Cancer Biology, Vanderbilt University Medical Center, Nashville, TN, United States

**Keywords:** allogeneic hematopoietic cell transplantation, GvHD, T cells, glycolysis, GVL effects, metabolism

## Abstract

Alloreactive donor T cells undergo extensive metabolic reprogramming to become activated and induce graft-versus-host disease (GVHD) upon alloantigen encounter. It is generally thought that glycolysis, which promotes T cell growth and clonal expansion, is employed in this process. However, conflicting data have been reported regarding the requirement of glycolysis to induce T cell-mediated GVHD due to the lack of T cell-specific treatments using glycolysis inhibitors. Importantly, previous studies have not evaluated whether graft-versus-leukemia (GVL) activity is preserved in donor T cells deficient for glycolysis. As a critical component affecting the clinical outcome, it is necessary to assess the anti-tumor activity following treatment with metabolic modulators in preclinical models. In the present study, we utilized T cells selectively deficient for glucose transporter 1 (Glut1^T-KO^), to examine the role of glycolysis exclusively in alloreactive T cells without off-targeting effects from antigen presenting cells and other cell types that are dependent on glycolysis. We demonstrated that transfer of Glut1^T-KO^ T cells significantly improved acute GVHD outcomes through increased apoptotic rates, impaired expansion, and decreased proinflammatory cytokine production. In addition to impaired GVHD development, donor Glut1^T-KO^ T cells mediated sufficient GVL activity to protect recipients from tumor development. A clinically relevant approach using donor T cells treated with a small molecule inhibitor of glycolysis, 2-Deoxy-D-glucose ex vivo, further demonstrated protection from tumor development. These findings indicate that treatment with glycolysis inhibitors prior to transplantation selectively eliminates alloreactive T cells, but spares non-alloreactive T cells including those that protect against tumor growth. The present study has established a definitive role for glycolysis in acute GVHD and demonstrated that acute GVHD can be selectively prevented through targeting glycolysis.

## Introduction

Allogeneic hematopoietic stem cell transplantation (allo-HSCT), which provides donor T cell-mediated protection known as graft-versus-leukemia (GVL) effect, is a critical curative option for many types of hematologic malignancies (1, 2). However, donor T cells that recognize recipient alloantigens can also contribute to graft-versus-host disease (GVHD), the primary cause of non-relapse mortality (3). Nonspecific treatments such as T cell depletion therapies and broad immunosuppressants are linked to elevated rates of relapse and opportunistic infections (4–6). Novel approaches are therefore necessary for selectively targeting alloreactive T cells to preserve non-alloreactive T cells that mediate anti-tumor immunity.

The current understanding in T cell function is tightly linked to the metabolic state, prompting the use of metabolic modulation to dampen harmful inflammatory responses. Naïve, memory T cells and effector T cells adopt distinct metabolic profiles to support survival and functional requirements. Previous studies explored manipulation of T cell bioenergetics to dampen the inflammatory response as a result of increased dependence on oxidative phosphorylation (OXPHOS) (7). In the current study, we investigate the potential of targeting a different metabolic pathway, glycolysis, in acute GVHD mediated by donor T cells. In contrast to naïve and memory T cells, activated effector T cells become highly dependent on aerobic glycolysis to fulfill biosynthetic demands for cell growth and division (8, 9), cytokine production (10, 11), which promote pathogenic T cell responses in various inflammatory conditions (12–16). Despite the large body of studies, the role of glycolysis in the pathogenicity of alloreactive T cells and the sparing of GVL activity with glycolysis blockade remain poorly understood (7, 17–20). Previous studies indicated that alloreactive T cells activated *in vivo* are primarily dependent on OXPHOS and fatty acid oxidation (FAO) (17, 18). In contrast, *in vivo* studies by Nguyen et al. showed that alloreactive T cells preferentially utilize glycolysis through phenotypic analysis (20). However, these studies could not exclude the dependence of antigen presenting cells (APCs) on glycolysis due to the systemic treatment with glycolysis inhibitors (20–23). Non-specific treatments using metabolic inhibitors can affect the function and survival of other cell types and cannot be assumed to accurately reflect the biology of alloreactive T cells. Other groups also demonstrated indirect connections between glycolysis and T cell-mediated GVHD (24, 25). More importantly, whether glycolysis inhibition is capable of preserving anti-tumor effects of non-alloreactive T cells is unknown. It is imperative to evaluate GVL effects in preclinical studies to prevent tumor relapse prior to the introduction of glycolysis inhibitors to the clinical setting.

A model limiting the utilization of glycolysis exclusively in T cells is necessary to address its role in T cell-driven GVHD and the preservation of GVL effects. Glucose uptake in T cells can be facilitated through glucose transporter (Glut) family members Gluts 1, 3, 6, and 8 (15). Glut1, the primary glucose transporter in T cells, is upregulated as soon as 2 hours following activation (15). Transgenic animals that constitutively express Glut1 are susceptible to the development of systemic inflammatory diseases (26, 27). Given the discordant findings (7, 17, 18, 20), we previously utilized animals harboring a T cell-specific genetic deletion for Glut1 (Glut1^T-KO^) to address the role of glycolysis in GVHD (15). However, whether the effect on disease progression is a strain-specific phenomenon, the mechanisms leading to the differences in GVHD development, and impacts on GVL effects were not examined. Donor T cells derived from these animals are functionally deficient for glycolysis, allowing for the examination of glycolysis in T cell-mediated GVHD. In the current study, we examined the molecular pathways by which glycolysis modified the pathogenic phenotype of alloreactive T cells through proliferative response and cell death mechanisms, demonstrating a key role for glycolysis without confounding factors from other glycolysis-dependent cell types (21–23). We also evaluated for the first time the therapeutic potential and feasibility for the separation of GVL from GVHD through ex vivo glycolysis inhibition using the small molecule inhibitor, 2-Deoxy-d-glucose (2-DG).

## Methods

### Mice

C57BL/6 (H-2^b^, CD45.2), C3H/HeJ (H-2^k^, CD45.2), BALB/c (H-2^d^, CD45.2), B6.SJL (H-2^b^, CD45.1) mice were purchased from Jackson laboratories (Bar Harbor, ME). Glut1^T-KO^ (Glut1^fl/fl^ x CD4^Cre^) mice, Glut1^fl/fl^ mice, and TCR-tg 4C mice are in the C57BL/6 background as described previously (15, 28–30). Wild-type (WT) animals include both C57BL/6 and littermate controls. All mice were maintained in a specific pathogen-free facility at Duke University. All experimental procedures were approved by the Institutional Animal Care and Use Committee (IACUC) of the Duke University Medical Center.

### Tumor Cell Lines

Luciferase (Luc)- and the enhanced green fluorescent protein (EGFP)-expressing (Luc-EGFP) BCL1 cells, a B-cell leukemia/lymphoma cell line of BALB/c origin, were a generous gift from Dr. Defu Zeng (City of Hope, Duarte, CA). A20 cells, another B-cell leukemia/lymphoma cell line of BALB/c origin, were initially purchased from ATCC (Manassas, VA). A20 cells expressing the Luc-EGFP gene were made by lentivirus-mediated gene transduction. Briefly, 293T cells cultured in Dulbecco’s Modified Eagle Medium (DMEM) media (Sigma-Aldrich, St. Louis, MO, USA) were co-transfected with pLEX (ThermoFisher)-EF1a-luciferase-EGFP together with the packaging plasmids, pMD2.G (a gift from Didier Trono (Addgene plasmid # 12259) and psPAX2 (A gift from Didier Trono (Addgene plasmid # 12260)), by calcium phosphate precipitation. After 24 hours, the DMEM media was replaced with fresh medium. At 48 hours after transfection, medium containing lentivirus was harvested and filtered through a 0.45 µM syringe filter. Viral infection was carried out in a 12-well plate using 5 × 10^5^ A20 cells with 0.5 ml of lentiviral medium containing 10 µg/mL polybrene (Sigma-Aldrich, St. Louis, MO). At 24 hours after infection, cells were selected with 1 µg/mL puromycin for 7 days and clonal Luc-EGFP positive cells were then selected by FACS sorting. Periodically, cells were treated with puromycin to weed out cells which had silenced reporter gene expression.

### Murine Cell Preparation and T Cell Stimulation

Murine total, CD4^+^, or CD8^+^ T cells were isolated from splenocytes by negative selection using mouse Pan T Cell Isolation Kit II (Miltenyi, Germany). Dendritic cells (DCs) were isolated from splenocytes using CD11c Microbeads UltraPure (Miltenyi). Bone marrow cells were collected from femurs and tibia by flushing using a syringe and passing through a strainer. To prepare T cell depleted bone marrow (TCDBM), bone marrow cells were first incubated with anti-CD90.2 antibody (clone 30H12; BD Pharmingen, CA) on ice for 1 hour. Subsequently, cells were treated with Low Tox-M Rabbit Complement (Cedarlane, Burlington, Canada) for 1 hour at 37°C and washed twice for injection. For *in vitro* T cell stimulation, 7.5 x 10^5^ T cells isolated from donor spleens were incubated in 12 wells, flat-bottomed plates with 1.5 x 10^5^ BALB/c irradiated DCs (20 Gy) at 37°C in 5% CO_2_ for 16 hours; irradiated BALB/c splenocytes (20 Gy) were used when indicated. T cells isolated from the recipient spleens were utilized for *in vivo* expansion analyses. For antibody stimulation *in vitro*, 12 wells, flat-bottomed plates were coated with goat anti-hamster IgG antibody (Invitrogen) at 20ug/ml overnight, followed by wash with PBS prior to stimulation with anti-CD3 at 1ug/ml (BD Pharmingen, clone 145-2C11) and anti-CD28 at 0.3ug/ml (Invitrogen, clone 37.51) antibodies. For metabolic assays, T cells were co-cultured with BALB/c irradiated DCs or IL-7 (0.3 ng/ml) for 120 hours. For intracellular staining of TNFα, WT or Glut1^T-KO^ T cells were stimulated with purified BALB/c DCs for 72 hours, with the addition of PMA (Sigma, 20ng/ml), ionomycin (Sigma, 1uM), and monensin (Thermofisher) 4 hours prior to collection. For ex vivo inhibition assays, 1 x 10^6^ T cells were first stimulated with irradiated BALB/c splenocytes (20 Gy) for 16 hours in complete RPMI with 10% fetal bovine serum. Following 16 hours, T cells were washed and stimulated with freshly isolated BALB/c splenocytes (irradiated) for an additional 24 hours, 48 hours, 72 hours, or 96 hours in the presence of media control or 2-DG at a final concentration of 8mM.

### Human Cell Preparation and T Cell Stimulation

Human T cells were purified using RosetteSep human T cell enrichment cocktail (STEMCELLTechnologies, Vancouver, Canada) from donor peripheral blood mononuclear cells (PBMCs). T cells (1.25 x 10^5^ cells) were co-cultured with irradiated PBMCs (20 Gy) from unrelated donors (5 x 10^5^ cells) for 16 hours, followed by 24-hour incubation with 2-DG, washed and incubated with PBMC stimulators or Dynabeads human T-Activator CD3/CD28 for 72 hours (Thermo Fisher, Waltham, MA). Human samples from de-identified healthy donors were obtained from American Red Cross under an approved protocol.

### GVHD Model

Recipient mice were lethally irradiated at 9.5 Gy for C3H/HeJ, 8.5 Gy for BALB/c, or 10.5 Gy for C57BL/6 mice using a Mark I-68A ^137^Cs irradiator (JL Shepherd and Associates, San Fernando, CA) and transplanted *via* tail vein injection within 4 hours following irradiation. Recipients were transplanted with 1 x 10^7^ TCDBM cells/mouse from C57BL/6 donors with or without 1 x 10^6^ T cells from WT or Glut1^T-KO^ mice. Survival, weight change, skin changes (hair loss and ruffling, erythema), hunching posture, diarrhea, and activity were monitored daily for clinical grading. Mice that met humane endpoints were sacrificed according to Duke University IACUC protocols.

### GVL Model

Recipient BALB/c mice were lethally irradiated at 8.5 Gy, followed by transplantation with 1 x 10^7^ TCDBM cells/mouse from C57BL/6 donors with or without T cells from WT or Glut1^T-KO^ mice, along with 5 x 10^5^ Luc-EGFP BCL1 cells or 1 x 10^5^ Luc-EGFP A20 cells. Survival and weight loss were recorded daily. Recipients were further monitored for tumor growth by bioluminescent imaging (BLI) and GVHD evidence by skin changes, activity, posture, and diarrhea. Biopsies were taken from spleen and liver for evidence of tumor growth. Mortality due to GVHD or tumor was distinguished by BLI, necropsy, and histology. In the absence of tumor detection, the cause of death was ruled as GVHD.

### Bioluminescent Imaging

Mice were anesthetized using isofluorane, followed by D-Luciferin injection (50 mg/kg, PerkinElmer, CT) 10 minutes prior to imaging. Imaging was performed using a Xenogen IVIS 100 imaging system (Xenogen Corporation, Alameda, CA) for maximal signal intensity at 5-minute exposure time. Living Image 2.5 software (Caliper, Newton, MA) was used for imaging analyses.

### Histologic Analysis

Biopsy samples were taken from skin, small and large intestines, liver, and spleen and were stored in neutral buffered formalin. Specimens were embedded in paraffin, cut into 5-μm sections, and stained with hematoxylin-eosin (H&E). Coded slides were assessed by D.C. single blinded to the GVHD status. Histological GVHD was graded using a semi-quantitative system based on histologic changes in the small intestine, colon, skin, and liver. Histological characteristics used for scoring included inflammatory infiltrates, apoptosis of keratinocytes, separation of dermal-epidermal junction, and formation of cleft, follicular dropout, and fibrosis in the skin; inflammation, apoptosis of bile duct epithelial cells, apoptosis of hepatocytes, cholestasis, fibrosis, and parenchyma in the liver; and lamina propria inflammatory cell infiltrate, crypt regeneration, crypt epithelial cell apoptosis, crypt loss, mucosal ulceration, and fibrosis in the intestine (31).

### Mixed Lymphocyte Reaction

Purified T cells (2.5 x 10^5^ cells) were incubated in 96-wells, flat-bottomed plates with 5 x 10^5^ irradiated (20 Gy) BALB/c splenocytes for indicated periods at 37°C in 5% CO_2_. Cells were pulsed with ^3^H-thymidine (1μCi [0.037MBq]/well) 16 hours before being counted by a MicroBeta Trilux liquid scintillation counter (EG&G Wallac, Turku, Finland).

### Metabolic Assays

Extracellular acidification rate (ECAR) and oxygen consumption rate (OCR) assays were performed using the XF24 extracellular flux analyzer (Seahorse Bioscience) as previously described (32). ECAR was measured at indicated time points following sequential compound injections (10 mM glucose, 1 µM oligomycin, and 20 mM 2-DG). Basal OCR was measured prior to compound injection. Glucose uptake assays were described previously (Wieman et al., 2007). 2-Deoxy-d-[H^3^] glucose (2 μCi/reaction) was added to T cell cultures and quenched by 200 µM phloretin (Calbiochem, San Diego, CA). Radioactivity of solubilized cell pellets was measured using a scintillation counter.

### Enzyme-Linked Immunosorbent Assay

Supernatants from T cell cultures were collected and assessed by enzyme-linked immunosorbent assay (ELISA) using antibodies against interferon γ (IFNγ) and interleukin-2 (IL-2) (BD Pharmingen, San Jose, CA) as described previously (26).

### Flow Cytometry

The following antibodies were used to detect surface protein expression: anti-CD4-PE (clone H129.19), anti-CD4-APC (clone RM4-5), anti-CD8-PE-Cy7 (clone 53-6.7) were purchased from BD Pharmingen (San Diego, CA) and BD Biosciences (Franklin Lakes, New Jersey); anti-CD69-PerCP-Cy5.5 (clone H1.2F3) was purchased from Biolegend (San Diego, CA). Fixable Viability Dye eFluor 780 (catalog 65-0865) was used to distinguish viable cells (eBioscience, San Diego, CA). For intracellular staining, anti-pS6-PE (eBioscience, clone cupk43k) and anti-Bim-PE (CST, Danvers, MA, clone C34C5) were used; anti-Mcl-1 (clone Y37) and anti-Noxa (clone 114C307) primary antibodies were purchased from Abcam (Cambridge, UK). Secondary antibodies, anti-rabbit IgG Fab2-AF647 (catalog ab181347) and anti-mouse IgG Fab2-AF647 (catalog ab169358) were purchased from Abcam. For intracellular staining, cells were first stained with antibodies for surface proteins, fixed with 4% PFA, then permeabilized using 0.5% Tween 20 in PBS. For staining of TNFα, eBioscience Foxp3/Transcription Factor Staining Buffer Set was used (ThermoFisher). Cells were fixed and permeabilized at 4 degrees Celsius for 30 minutes, followed by washing with 1x permeabilization buffer, intracellular staining for 20 minutes. Following staining, cells were washed twice before running. For secondary staining, secondary antibodies were added following addition of unconjugated primary antibodies. For isotype controls, Rabbit IgG XP (R)-PE (CST, catalog 5742S), mouse IgG1k-PE (BD Pharmingen, clone MOPC-21), Rabbit IgG (Abcam, ab37415), mouse IgG1k (Abcam, clone B11/6) were used. For apoptosis assay, the Apoptosis Detection Kit (BD Pharmingen), which includes Annexin V-PE and 7-Amino-Actinomycin D (7AAD), was used. Stained samples were analyzed using FACSCanto Flow Cytometer (BD Biosciences) and data were analyzed using FlowJo software (Tree Star, Ashland, OR).

### Western Blotting

Cells were lysed with Pierce™ IP Lysis Buffer (ThermoFischer), which contains 25mM Tris-HCl pH 7.4, 150 mM NaCl, 1% NP-40, 1 mM EDTA, 5% glycerol, supplemented with protease inhibitor (Thermo Scientific) and phosphatase inhibitor (Thermo Scientific). Cell debris was then removed by spinning for 5 minutes at 4°C. Protein concentrations were determined using the Pierce BCA Protein Assay Kit (Thermo Scientific). Whole cell extracts (50μg of proteins) were fractionated by SDS-PAGE and transferred to a nitro cellular membrane using a transfer apparatus according to manufacturer’s instructions (Bio-Rad). Membranes were blocked with LICOR blocking buffer, washed and incubated with primary antibodies (1:1000 in blocking buffer) at 4°C for 12 hours. After washing, membranes were incubated with a 1:10000 dilution (in blocking buffer) of fluorescent 700 or 800 anti-rabbit or anti-mouse antibodies for 1 hour at room temperature. Blots were washed with TBST five times and scanned using LICOR machine. Anti-Puma antibody (ab9643), anti-Noxa antibody (clone 114C307, ab13654), and anti-Mcl-1 antibody (clone Y37, ab32087) were purchased from Abcam. Anti-Mdm2 antibody (clone D-7, sc-13161) was purchased from Santa Cruz Biotechnology Inc. (Dallas. TX).

### Statistics

Data were analyzed using Prism Graphpad (Version 6, San Diego, CA). Error bars represent mean ± SEM. Unpaired two-tailed student’s *t* tests, one-way ANOVA with Tukey’s multiple comparisons test, were utilized for group comparisons. Survival curve comparisons were performed using Log-Rank (Mantel-Cox) test. P-values < 0.05 were considered statistically significant.

## Results

### Glut1 Is Required for Donor T Cells to Induce GVHD

**Figure 1:**
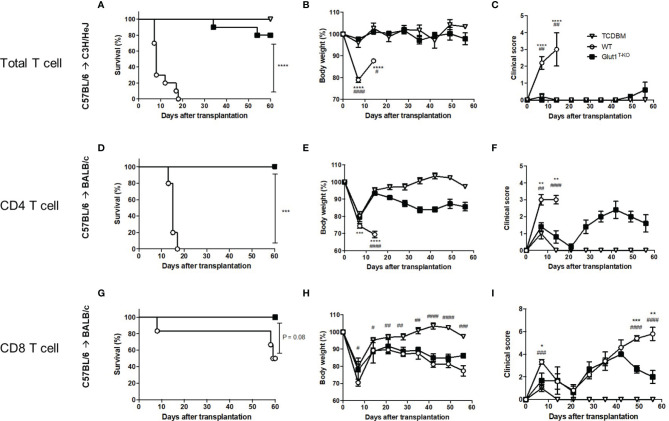
Glut1 is required for donor T cells to induce acute GVHD. Acute GVHD was induced by transplantation of C57BL/6-derived 1 x 10^7^ TCDBM or along with 1 x 10^6^ WT or Glut1^T-KO^ total T cells into lethally irradiated (9.5 Gy) C3H/HeJ recipients **(A–C)**. Lethally irradiated (8.5 Gy) BALB/c recipients were transplanted with 1 x 10^6^ WT or Glut1^T-KO^ CD4^+^ **(D–F)** or CD8^+^ T cells **(G–I)**, along with 1 x 10^7^ TCDBM from C57BL/6 donors. Recipients were monitored for survival **(A, D, G)**, body weight **(B, E, H)**, clinical score **(C, F, I)** up to 56 days after transplantation. Data were representative of three experiments. ****P < 0.0001, ***P < 0.001, log-rank test [n = 10 per group, A; n = 5 per group, (**D, G**)]; data are shown as mean ± SEM **(B, C, E, F, H, I)**, *P < 0.05, **P < 0.01, ***P < 0.001, ****P < 0.0001 (Glut1^T-KO^ vs. WT); ^#^P < 0.05, ^##^P < 0.01, ^###^P < 0.001, ^####^P < 0.0001(TCDBM vs. WT), 2-tailed Student t test.

We previously demonstrated the role of Glut1 in T cell-mediated acute GVHD using the C57BL/6 → BALB/c major histocompatibility complex (MHC)-mismatched bone marrow transplant (BMT) model (15). To confirm that the observation is not strain-specific, C3H/HeJ recipients were also utilized for the transfer of Glut1^T-KO^ or wild-type (WT) C57BL/6 T cells to induce acute GVHD. All WT recipients died from GVHD within 20 days while eight out of ten Glut1^T-KO^ T cell recipients survived long-term (**Figure 1A**). In addition, Glut1^T-KO^ T cell recipients showed comparable body weight and clinical scores with TCDBM recipients (**Figures 1B, C**). Therefore, consistent with the previous study, these findings further support a key role for Glut1 to promote donor cell pathogenicity.

We further examined whether Glut1 is required for CD4^+^ and CD8^+^ T cells to induce acute GVHD, respectively. In contrast to WT recipients, both CD4^+^ Glut1^T-KO^ and CD8^+^ Glut1^T-KO^ T cell recipients survived long-term (**Figures 1D, G**). However, the kinetics of GVHD development and target organs affected differed. Both body weights and clinical scores in CD4^+^ Glut1^T-KO^ T cell recipients significantly improved early following BMT (**Figures 1D–F**). In contrast, the kinetics of GVHD development in CD8^+^ T cell recipients is relatively delayed, leading to improvement in Glut1^T-KO^ T cell recipients later during disease progression compared to the control group (**Figures 1G–I**). Target organ damage was also assessed by histology (**Figures S1, S2**). Both small intestine and colon exhibited reduced damage in CD4^+^ Glut1^T-KO^ compared to WT recipients (**Figures S1A, S2A**). In contrast, skin damage was significantly reduced in CD8^+^ Glut1^T-KO^ recipients (**Figures S1B, S2B**). Overall, transfer of either CD4^+^ or CD8^+^ Glut1^T-KO^ T cells significantly improved long-term survival and ameliorated acute GVHD.

### Glut1 Is Required for the Metabolic Reprogramming and Expansion of Alloreactive T Cells

**Figure 2:**
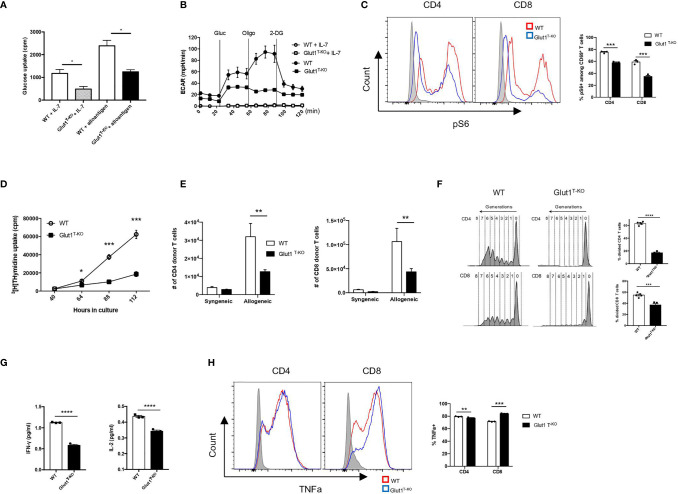
Glut1 mediates the expansion of alloreactive T cells. WT or Glut1^T-KO^ T cells were stimulated with BALB/c dendritic cells. After 5 days in culture, glucose uptake was measured **(A)**. ECAR was assessed with the addition of glucose (gluc), oligomycin (olig), and 2-DG **(B)**. WT or Glut1^T-KO^ T cells were stimulated for 16 hours using BALB/c DCs and analyzed by flow cytometry for phospho-S6 levels in CD69^+^ T cells **(C)**. Expansion *in vitro* was measured by ^3^H-TdR incorporation assay at indicated time points after culture **(D)**. Expansion *in vivo* was measured in T cells isolated from the spleen seven days after transplantation with 1 x 10^7^ TCDBM from B6.SJL donors and 1 x 10^6^ WT or Glut1^T-KO^ T cells on the C57BL/6 background into BALB/c recipients **(E)**. T cell proliferation following isolation from the spleen was analyzed (left panel) and measured by frequency (right panel) 58 hours following transfer of CellTrace Violet (CTV)-labelled T cells along with B6.SJL TCDBM into BALB/c recipients **(F)**. IFNγ and IL-2 production were assayed by ELISA using supernatants from T cell cultures **(G)**. **(H)** WT or Glut1^T-KO^ T cells were stimulated with purified BALB/c DCs for 72 hours, with the addition of PMA (20ng/ml), ionomycin (1uM), and monensin 4 hours prior to intracellular staining for TNFα. Data are representative of two (F, n = 3) or three experiments (n = 3, A-D, and G; n = 5, E and F) and are shown as mean ± SEM **(B–F)**. *P < 0.05, **P < 0.01, ***P < 0.001, ****P < 0.0001 (Glut1^T-KO^ vs. WT), 2-tailed Student t test.

T cells rapidly undergo metabolic reprogramming following activation, prioritizing glucose metabolism to promote growth and proliferation (33). We first assessed whether alloreactive Glut1^T-KO^ T cells were able to initiate metabolic reprogramming. Glut1^T-KO^ T cells had significantly decreased glucose uptake following alloantigen stimulation (**Figure 2A**). Alloreactive Glut1^T-KO^ T cells were unable to utilize glycolysis, indicated by ECAR compared to WT T cells during glycolysis stress test (**Figure 2B**). Metabolic assays further confirmed deficient glycolysis (**Figure S2A**), glycolytic capacity (**Figure S2B**), and glycolytic reserve (**Figure S2C**) of alloreactive Glut1^T-KO^ T cells compared to control. These results suggest that Glut1^T-KO^ T cells display overall significant defects in glucose uptake and glycolytic metabolism upon alloantigen challenge.

Mammalian target of rapamycin complex 1 (mTORC1) regulates glucose metabolism through HIF1α and c-Myc to support biosynthesis and proliferation (33, 34). Although rapamycin has been shown to dampen GVHD by inhibiting glycolysis (20), it is unclear whether glucose availability modulates mTORC1 activity to regulate alloreactive T cell response. We hypothesize that mTORC1, a nutrient sensor (34), responds to glucose availability to modulate donor cell pathogenicity. Alloreactive T cells positive for CD69 expression (**Figures S3A** and **S3B**) were assessed for the phosphorylation status of the small ribosomal subunit S6 (pS6), a downstream target for mTORC1 signaling. Phosphorylation of S6 (Ser235/236) in resting Glut1^T-KO^ T cells was significantly lower than WT T cells (**Figure 2C**). Following stimulation, Glut1^T-KO^ T cells demonstrated profoundly decreased phospho-S6 levels (**Figure 2C**). Glucose availability therefore leads to sustained mTORC1 activation in alloreactive T cells.

To determine the requirement for T cell expansion, tritium thymidine uptake was assessed in MLR. Glut1^T-KO^ T cells displayed drastically impaired thymidine uptake as early as 64 hours following stimulation (**Figure 2D**). To test whether glycolysis is required for *in vivo* expansion upon alloantigen encounter, T cells were transferred into irradiated allogeneic or syngeneic recipients. While expansion in syngeneic recipients did not differ, Glut1^T-KO^ T cells exhibited significantly impaired capacity to undergo expansion compared to WT T cells in allogenic recipients (**Figure 2E**). Furthermore, Glut1^T-KO^ T cells failed to undergo robust proliferation, indicated by the lack of subsequent generations following divisions (**Figure 2F**). Similar defects were observed in CD69^+^ alloreactive Glut1^T-KO^ T cells (**Figures S4A, S4B**). Glycolysis has also been linked to cytokine production through the sequestration of cytokine transcripts (10). Expression of inflammatory cytokines IFNγ and IL-2 was measured in alloreactive T cells. Glut1^T-KO^ T cells displayed significantly reduced capacity to produce both cytokines compared to WT T cells (**Figure 2G**). TNFα expression was also assessed in WT and Glut1^T-KO^ T cells following 72 hours of stimulation with purified BALB/c DCs. While Glut1^T-KO^ CD4 T cells exhibited slightly reduced expression compared to WT group, Glut1^T-KO^ CD8 T cells demonstrated increased TNFα expression relative to WT T cells (**Figure 2H**). In summary, we demonstrated that glycolysis is indispensable for alloreactive T cell expansion and effector cytokine production, which cannot be rescued by OXPHOS in Glut1^T-KO^ T cells (**Figure S2D**).

### Glut1 Is Required for the Survival of Alloreactive T Cells

**Figure 3:**
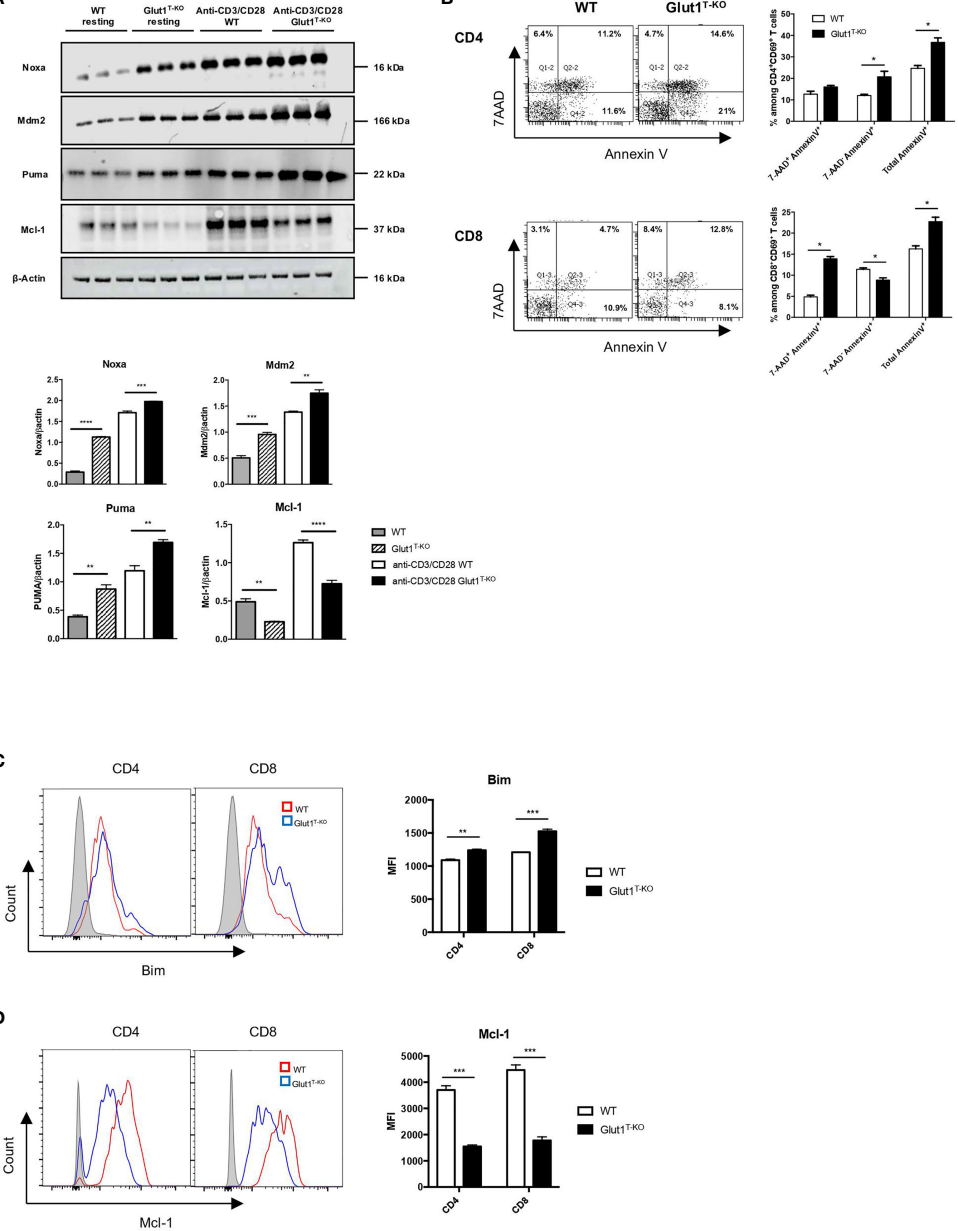
Glut1 modulates alloreactive T cell survival. **(A)** Freshly isolated WT or Glut1^T-KO^ T cells or those stimulated with anti-CD3 (1ug/ml) and anti-CD28 (0.3ug/ml) antibodies for 16 hours were assessed for the expression of Mdm2, Puma, Noxa, and Mcl-1 using Western blotting (upper panel). Results were quantified for fresh T cells and antibody-stimulated T cells (lower panel) (n = 3, one-way ANOVA with a Tukey’s multiple comparisons test). **(B)** WT or Glut1^T-KO^ T cells were stimulated for 16 hours by irradiated (20 Gy) BALB/c DCs and analyzed by flow cytometry for Annexin V and 7AAD. T cells were gated on CD69^+^ CD4^+^ or CD8^+^ T cells. Bim **(C)** and Mcl-1 **(D)** expression were evaluated. Data are representative of three experiments (n = 3) and are shown as mean ± SEM. *P < 0.05, **P < 0.01, ***P < 0.001, ****P < 0.0001, 2-tailed Student t test.

In addition to proliferation, alteration of survival signals is a potential modulator of pathogenicity. Glut1 expression has been shown to support resting T cell survival through the stabilization of pro-survival factors (35). To determine whether the apoptotic pathway is involved in regulating viability in response to glucose metabolism, we assessed the expression of various candidate proteins 16 hours following activation by anti-CD3 and anti-CD28 antibodies. Proteins linked to the apoptotic pathway, including Mdm2, Puma, and Noxa, were drastically increased in activated Glut1^T-KO^ T cells relative to WT control (**Figure 3A**). By contrast, the anti-apoptotic protein Mcl-1 was significantly upregulated compared to WT T cells (**Figure 3A**).

We further assessed whether glycolysis is also involved in regulating cell survival in alloreactive T cells. Viability analysis demonstrated significantly less live Glut1^T-KO^ T cells compared to WT T cells following alloantigen stimulation *in vitro* (**Figure S5A**). Apoptosis was subsequently assessed using Annexin V and 7AAD staining. While both CD4^+^ and CD8^+^ alloreactive Glut1^T-KO^ T cells underwent increased apoptosis compared to WT T cells, the apoptosis kinetics differed. CD8^+^ CD69^+^ Glut1^T-KO^ T cells appeared to undergo apoptosis earlier than CD4^+^ CD69^+^ T cells inferred from percentages of Annexin V^+^ cells (**Figure 3B**).

Regulation of pro-apoptotic and anti-apoptotic protein expression can alter the survival outcome in response to cellular stress. Impaired glucose metabolism can lead to apoptosis in response to endoplasmic reticulum (ER) stress mediated by Bim, a pro-apoptotic Bcl-2-family protein (36). Bim expression is higher in Glut1^T-KO^ T cells compared to WT T cells in both freshly isolated state and activated state (**Figures S5B** and **3C**). Though the demand for glycolysis is lower in resting T cells compared to activated T cells, a minimal rate of glycolysis is still required to meet basal energy demands (26), potentially contributing to the difference in baseline Bim expression. Regulation of anti-apoptotic Bcl-2 proteins such as Mcl-1 can also regulate survival (35, 37) Alloantigen-stimulated Glut1^T-KO^ T cells failed to provide adequate survival signal through Mcl-1 compared to WT T cells (**Figure 3C**). Differences in Mcl-1 expression were readily detected in both anti-CD3 and anti-CD28 antibody activated T cells and alloreactive T cells (**Figures 3A, D**). Baseline differences in Mcl-1 expression between WT and Glut1^T-KO^ T cells were detectable using Western blots (**Figure 3A**) but not flow cytometry (**Figure S5C**), which can be attributed to variation in detection sensitivity between methods of detection. In addition, the balance of Mcl-1 and Noxa, a BH3-only pro-apoptotic factor and a binding partner for Mcl-1, can be regulated by glucose availability (38). Since Noxa expression was reduced in the presence of both anti-CD3 + anti-CD28 antibodies as well as alloantigens (**Figures 3A** and **S5D**), the skewed Noxa/Mcl-1 ratio may render Glut1^T-KO^ T cells more prone to apoptosis.

### Glut1 Deficiency in Donor T Cells Ameliorates GVHD While Preserving GVL Effects

It is crucial for GVHD treatments to selectively inhibit alloreactive T cells without compromising the GVL effect. To test the effect of glycolysis inhibition on GVL preservation, lethally irradiated BALB/c recipients were engrafted with WT or Glut1^T-KO^ T cells, TCDBM, accompanied by challenge with BCL1 cells, a BALB/c-derived leukemia/lymphoma cell line.

**Figure 4:**
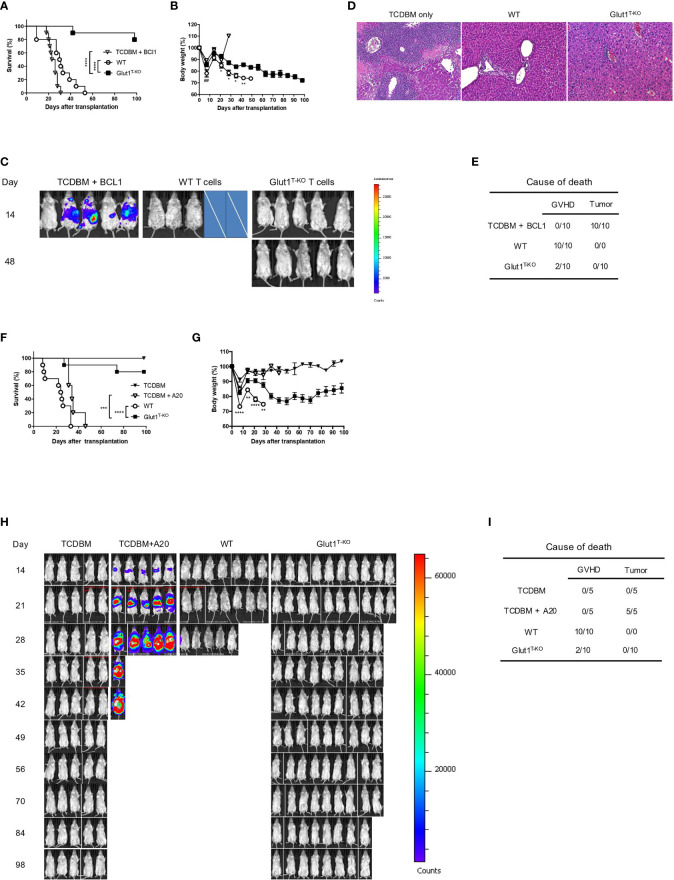
Transfer of Glut1^T-KO^ T cells inhibits GVHD development and spares GVL activity. Lethally irradiated (8.5 Gy) BALB/c recipients were transplanted with 1 x 10^6^ WT or Glut1^T-KO^ T cells, along with 1 x 10^7^ TCDBM and 5 x 10^5^ BCL1 cells. Recipients were monitored for survival **(A)** and body weight **(B)** up to 100 days after transplantation. Development of leukemia/lymphoma **(C)** was monitored by BLI. Cross symbols indicate death prior to BLI. H&E histology (10x & 40x) of liver **(D)** from recipients was assessed (time at sample collection: TCDBM + BCL1, day 21; WT, day 27; Glut1^T-KO^, day 105). Cause of death due to GVHD or tumor development was summarized **(E)**. **(F)** Lethally irradiated (8.5 Gy) BALB/c recipients were transplanted with 1 x 10^6^ WT or Glut1^T-KO^ T cells, along with 1 x 10^7^ TCDBM and 1 x 10^5^ A20 cells. Recipients were monitored for survival **(F)** and body weight **(G)** up to 100 days after transplantation. Development of leukemia/lymphoma **(H)** was monitored by BLI. Cause of death due to GVHD or tumor development was summarized **(I)**. ***P < 0.001, ****P < 0.0001, log-rank test **(A, F)**; *P < 0.05, **P < 0.01, ****P < 0.0001 (Glut1T-KO vs. WT); ^##^P < 0.01 (TCDBM vs. WT), 2-tailed Student t test **(B, G)**. Data are representative of three experiments (n = 10 per group, 1 x 10^6^ T cell recipients).

TCDBM + BCL1 group succumbed to tumor challenge within 31 days following transplantation (**Figures 4A, B**), indicated by BLI (**Figure 4C**). Histology analysis further confirmed metastatic invasion of the liver parenchyma, indicated by enlarged and hyperchromatic nuclei of neoplastic cells (**Figure 4D**). While Glut1 deficiency did not completely protect recipients from GVHD as evidenced by gradual weight loss (**Figure 4B**), analyses of target organ histology indicated lower pathological scores in the skin and large intestine (**Figures S6**). Furthermore, transfer of 1 x 10^6^ Glut1^T-KO^ T cells significantly improved long term survival in majority of recipients compared to both TCDBM + BCL1 and WT T cell recipients, which all died from tumor or GVHD (**Figures 4A–E**). BLI analysis and necropsy revealed that Glut1^T-KO^ recipients remained tumor-free, demonstrating the preservation of GVL effects (**Figures 4C–E**). In contrast, all WT T cell recipients succumbed to GVHD within 100 days (**Figure 4E**). To confirm that the GVL effect of Glut1^T-KO^ T cells is not restricted to a specific tumor model, a second lymphoma cell line of BALB/c origin (A20) was used to evaluate protection against tumor development. Transfer of Glut1^T-KO^ T cells significantly improved survival compared to TCDBM + A20 and WT recipients (**Figure 4F**). While body weights of Glut1^T-KO^ recipients were lower than that of TCDBM control groups due to GVHD, they were significantly higher than WT recipients, demonstrating ameliorated GVHD development (**Figure 4G**). BLI analyses and necropsy showed that tumor growth was absent in all Glut1^T-KO^ recipients (**Figures 4H, I**), recapitulating protection against tumor using a different tumor model. A low dose of Glut1^T-KO^ T cells was also tested using the BCL1 tumor model and provided limited protection against tumor development (**Figure S7**), suggesting a role for glycolysis in GVL. However, this limitation can be overcome by increased dose of Glut1^T-KO^ T cells, indicating that glycolysis is not absolutely necessary for donor T cells to exert anti-tumor effects. Collectively, these data indicate that transfer of Glut1^T-KO^ T cells at sufficient concentrations is capable of preventing tumor growth and mortalities caused by GVHD, supporting the hypothesis that glycolysis targeting selectively inhibits alloreactive T cells.

### Inhibition of Glycolysis by 2-DG Selectively Targets Murine and Human Alloreactive T Cells *In Vitro*

**Figure 5:**
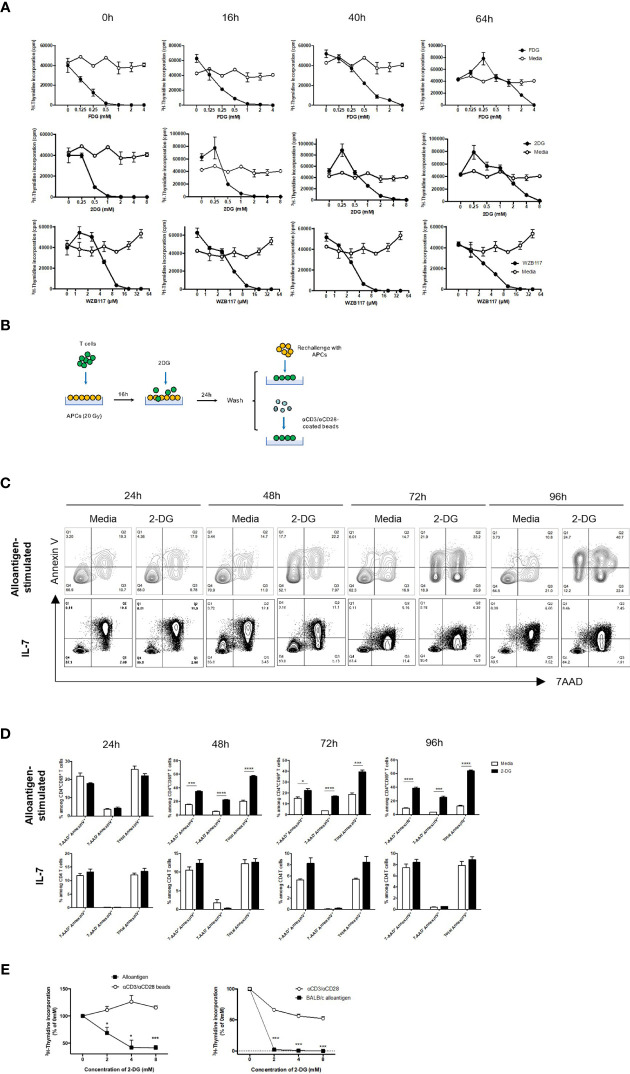
2-DG treatment selectively suppresses alloreactive T cells. WT or Glut1^T-KO^ T cells were stimulated for 0 hour, 16 hours, 40 hours, or 64 hours using irradiated (20 Gy) BALB/c splenocytes, followed by the addition of various concentrations of small molecule inhibitors and cultured for a total of 112 hours for the assessment of thymidine incorporation. T cell response was determined following the addition of FDG or media only (H_2_O), 2-DG or media only (H_2_O), WZB117 or media only (EtOH) **(A)**. Schematic diagram of T cells stimulated with irradiated MHC-mismatched APCs for 16 hours, followed by the addition of various concentrations of 2-DG, washed, then rechallenged with alloantigens or anti-CD3 and anti-CD28 antibodies **(B)**. WT T cells were first stimulated with irradiated (20 Gy) BALB/c DCs for 16 hours, followed by incubation with freshly isolated irradiated DCs in the presence of media control or 8mM 2-DG for 24-96 hours; WT T cells were cultured in IL-7 (10ng/ml) for 16 hours plus 24-96 hours, and analyzed for Annexin V and 7AAD **(C, D)**. T cells were gated on CD4^+^ CD69^+^ for alloantigen-stimulated samples and CD4^+^ for IL-7-treated samples. **(E)** The proliferative response of mouse (left panel) and human T cells (right panel) cultured according to **(B)** was measured by ^3^H-TdR incorporation assay. *P < 0.05, ***P < 0.001, ****P < 0.0001, 2-tailed Student t test; data are representative of two experiments (n = 3 per group).

A clinically-relevant approach for glycolysis inhibition to ameliorate GVHD has been previously explored, though systemic treatments (20) can induce toxicity in the brain and skeletal muscles (39–41). To improve treatment specificity, donor T cells can be treated ex vivo in the presence of recipient alloantigens. A panel of small molecule inhibitors were evaluated for inhibition of alloreactive T cell proliferation. Both glucose analogs, fludeoxyglucose (FDG) and 2-DG, remarkably suppressed donor T cell response following stimulation (**Figure 5A**). The same effect was observed for 2-DG when the T cell response was measured by the number of CD69-expressing activated T cells (**Figure S8A, S8B**). 2-DG also showed potent inhibitory effect on 4C CD4^+^ T cells bearing transgenic T cell receptors (TCR-tg) specific for BALB/c alloantigens (**Figures S8C, S8D**). The Glut1 inhibitor, WZB117, also inhibited alloresponse (**Figure 5A**). As 2-DG has been shown to dampen inflammatory T cell response and given its wide accessibility in clinical trials, 2-DG was selected for subsequent assays (42–45).

Incubation of recipient antigen-stimulated donor T cells with glycolysis inhibitors prior to BMT can spare non-alloreactive T cells, reducing toxicity to graft recipients and selectively suppressing alloresponse (**Figure 5B**). Incubation with 2-DG selectively triggered apoptosis in activated alloreactive T cells, indicated by a profound and consistent increase in AnnexinV^+^ populations and a corresponding decrease in the absolute number of alloreactive CD4 T cells (**Figures 5C, D**, upper panels). In contrast, 2-DG did not impact cell death outcomes in non-activated T cells treated with IL-7 (**Figures 5C, D**, lower panels). Similar findings were observed in alloantigen-stimulated versus IL-7-treated CD8 T cells (**Figure S9**). Following 2-DG incubation, secondary challenge with either alloantigens or anti-CD3- and anti-CD28-coated beads demonstrated that only alloresponse was significantly inhibited (**Figure 5E**). Importantly, as inhibition occurs exclusively during the ex vivo stimulation process, suppression of alloresponse by 2-DG prevents toxicity due to non-specific systemic treatments. Similarly, to test the efficacy in human T cells, purified donor T cells were first primed with irradiated PBMCs from irrelevant allogeneic donors, incubated with 2-DG, and followed by PBMC rechallenge or anti-CD3 and anti-CD28 antibody stimulation. Alloreactive responses underwent a dose-dependent reduction compared to non-specific stimulation, indicating that the proliferative capacity of alloreactive T cells is highly dependent on the ability of T cells to perform glycolysis (**Figure 5E**). Therefore, the optimal concentration of 2-DG at 8mM was utilized for subsequent *in vivo* assays.

### 2-DG-Mediated Inhibition of Glycolysis *Ex Vivo* Significantly Reduces GVHD While Preserving GVL Effects

**Figure 6:**
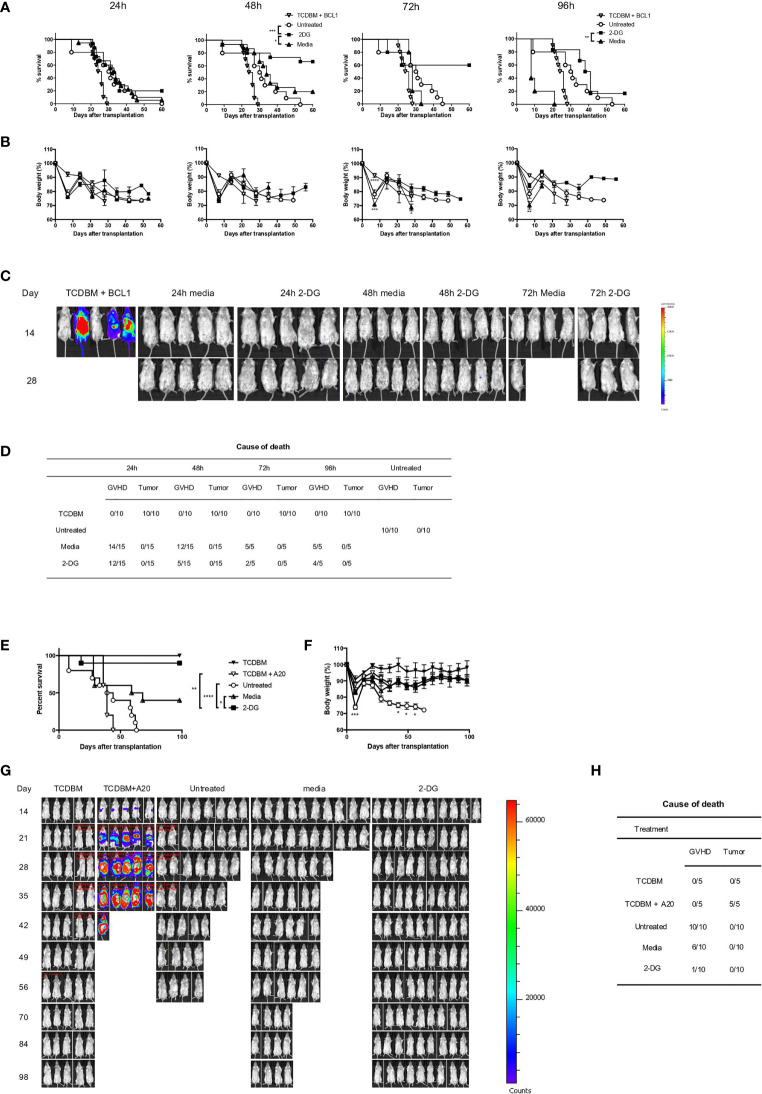
2-DG treatment ameliorates GVHD and preserves GVL effect. 1 x 10^6^ T cells from C57BL/6 donor spleens were first stimulated for 16 hours with irradiated BALB/c splenocytes, followed by addition of 8mM 2-DG or media control for indicated periods. T cells treated ex vivo or untreated control T cells at the same dose were transplanted into BALB/c recipients, along with 1 x 10^7^ TCDBM from C57BL/6 donors and 5 x 10^5^ BCL1 cells. Recipients were monitored for survival **(A)**, body weight **(B)**, and tumor growth in TCDBM and TCDBM + BCL1, 24-hour, 48-hour and 72-hour media control or 2-DG-treated groups. Tumor development was detected using BLI imaging on day 14 and day 28 following transplantation **(C)**. Cause of death due to GVHD or tumor development was summarized for different groups **(D)**. 1 x 10^6^ T cells treated as shown in **(A)** for 48 hours or untreated control T cells at the same dose were transplanted into BALB/c recipients, along with 1 x 10^7^ TCDBM from C57BL/6 donors and 1 x 10^5^ A20 cells. Recipients were monitored for survival **(E)**, body weight **(F)**, and tumor growth **(G)**. Cause of death was summarized for various groups **(H)**. *P < 0.05, **P < 0.01, ***P < 0.001, log-rank test; data are representative of two experiments (n = 15 per group, recipients for 24-hour and 48-hour ex vivo cultured T cells; n = 5, recipients for 72-hour and 96-hour ex vivo cultured T cells).

Given that Glut1^T-KO^ T cells preserved GVL effect and the promising *in vitro* data, we next tested the therapeutic potential of T cell-specific glycolysis inhibition using a clinically relevant model. Alloantigen-activated T cells were treated with 2-DG as shown in **Figure 5B** for 24-96 hours, followed by transfer into recipients along with TCDBM and BCL1 cells. Ex vivo inhibition for 24 hours demonstrated limited potency in GVHD prevention, while longer incubation periods (48-96 hours) with 2-DG significantly limited GVHD development without impairing GVL activity as demonstrated by survival, body weight, BLI tumor screening, and clinical scores (**Figures 6A–D** and **S10A, B**). Body weights of 72-hour-treated T cell recipients were significantly higher than those receiving media control T cells later following transplantation (**Figure 6B**). Interestingly, recipients for donor T cells treated with 2-DG for 48 hours gained an optimal survival advantage compared to those receiving uncultured and untreated T cells and those incubated with media control, resulting in the least amount of deaths by proportions caused by GVHD (**Figures 6A, D**). Protection against both GVHD and tumor development conferred by 48-hour-treated T cells was further assessed in a second tumor model using the A20 cell line. Ex vivo 2-DG treatment significantly improved survival compared to TCDBM + A20, WT T cell, and media control recipients (**Figure 6E**). Furthermore, transfer of 2-DG-treated T cells improved body weight compared to WT T cell recipients, as well as exhibiting reduced GVHD severity relative to both WT and media control recipients (**Figures 6F** and **S10C**). Together with BLI analyses and necropsy results (**Figures 6G, H**), we demonstrated that GVHD and tumor development can be attenuated using ex vivo 2-DG treatment. The results from these experiments provide further evidence that targeting glycolysis in alloantigen-specific T cells ex vivo preserves T cell response against irrelevant antigens, potentially providing protection against malignancies and opportunistic pathogens.

## Discussion

Activated T cells are dependent on aerobic glycolysis to support growth, division, and effector functions (9, 19, 46). Previous studies revealed conflicting results regarding the role of glycolysis in the pathogenesis of T cell-mediated GVHD (7, 15, 17, 18, 20). In the current study, we utilized T cells genetically deficient for Glut1 to directly demonstrate the requirement for glycolysis in donor T cell-mediated acute GVHD without affecting glycolysis in antigen presenting cells. We established that glycolysis modulates the magnitude of T cell response through proliferation and survival. We further demonstrated ex vivo glycolysis inhibition that specifically targets alloreactive T cells to prevent acute GVHD while sparing GVL effect as a preventive approach.

Several studies reported that elevated glucose metabolism is strongly associated with donor T cell-mediated GVHD (47, 48). Positron emission tomography studies revealed that glucose analog uptake is correlated with donor cell infiltration and GVHD symptoms (48). A retrospective single-cell RNA sequencing study identified upregulated genes encoding glycolytic enzymes in GVHD patients (47). Despite the phenotypic analyses and the existing paradigm for the dependence of activated T cells on glycolysis, other groups reported that alloreactive T cells *in vivo* are less glycolytic and are primarily dependent on oxidative metabolism, namely FAO (7, 17, 18). However, the absence of conditioning-associated tissue damage in these models contributes to limited release of inflammatory cytokines during the priming phase of T cells, subsequently altering the regulation of metabolic reprogramming and inflammatory response. Moreover, the evaluation of key glycolysis parameters and contribution of homeostatic expansion were not addressed. Contrary to this report, Nguyen et al. used a comprehensive metabolite analysis to demonstrate that alloreactive T cells derived from an irradiated model highly upregulate glycolysis compared to syngeneic recipients. The administration of glycolysis inhibitors in recipients alleviated disease progression. However, the systemic treatment led to off-target effects that compromised efficacy that could be attributed to organ toxicity (20). Functional studies that specifically target glycolysis in T cells are therefore necessary to address the role of glycolysis in GVHD pathogenesis. To this end, we utilized donor T cells genetically deficient for Glut1, a major glucose transporter in activated T cells. We showed that in contrast to WT T cells, Glut1^T-KO^ alloreactive T cells showed significantly impaired capacity to adopt aerobic glycolysis (**Figures 2A, B** and **S2A–S2C**). Transplant experiments using Glut1^T-KO^ T cells remarkably prevented acute GVHD-associated clinical traits and improved survival (**Figure 1**). Glycolysis is required for both CD4^+^ and CD8^+^ T cell-mediated pathogenesis as transfer of Glut1^T-KO^ CD4^+^ or CD8^+^ T cells showed significant increase in body weight, reduced clinical score and target organ damage compared to controls (**Figure 1**). However, disease kinetics and target organ damage differed due to difference in natural disease progression associated with the transfer of CD4^+^ versus CD8^+^ T cells, with rapid improvement of disease progression and alleviated gastrointestinal tract damage in CD4^+^ T cell recipients compared to delayed disease resolution and reduced skin damage in CD8^+^ T cell recipients. It was observed that the Glut1^T-KO^ T cell recipients in the C57BL/6 → BALB/c setting (**Figures 1D–I**, **4**) had not completely recovered their body weights when they were sacrificed after day +100. Even though we did not observe obvious signs of chronic GVHD at necropsy (data not shown) and histological analysis (**Figure S1, S6**), these observations do raise a question whether this strategy has any impact on chronic GVHD. More comprehensive analyses using chronic GVHD models will be needed to answer this question. Taken together, the above experiments demonstrated dramatically improved long-term survival compared to recipients of donor cells with intact glycolysis, providing solid evidence that glycolysis is selectively required for acute GVHD development.

mTORC1 activation has been shown to support cell growth, proliferation, and T_eff_ functions by promoting translation and anabolic metabolism (19, 49–51). Nguyen et al. showed that mTORC1-deficient T cells have reduced ability to induce GVHD, accompanied by a less glycolytic phenotype (20). However, it remains unclear whether the utilization of glycolysis directly promotes mTORC1 signaling in alloreactive T cells. As a sensor for metabolic cues, mTORC1 activity can be modulated by glucose availability (8). Consistently, the current study (**Figure 2C**) demonstrated impaired mTORC1 activation as a direct consequence of impaired glycolysis. Additionally, mTORC1 activity is negatively regulated by AMPK, an energy stress sensor that promotes catabolic pathways including FAO and OXPHOS (52).

Glycolysis has been implicated to support survival as well as antigen-specific expansion (15). We first assessed the impact on T cell expansion. *In vitro* experiments indicated a profound defect in clonal expansion of alloreactive T cells upon antigen stimulation (**Figure 2D**). This observation was recapitulated *in vivo* by the numbers of donor T cells recovered (**Figure 2E**) and percentages of divided donor cells using proliferation dye (**Figure 2F**). The difference in response was exclusively seen in allogeneic recipients but not syngeneic controls (**Figure 2E**), indicating that alloreactive T cells are dependent on glycolysis for proliferation. Modulation of inflammatory cytokine secretion is also a critical determinant of T cell pathogenicity. Previous studies demonstrated a role for glycolytic enzymes in the translational regulation of inflammatory cytokines by engaging/disengaging glycolysis upon TCR crosslinking (10, 11). Glycolysis inhibition has also been linked to diminished cytokine production in previous studies, where 2-DG was systemically delivered to BMT recipients (20). However, glycolysis is also required for DC maturation and migration (21–23). Proliferative T cell response can be severely impaired when activated by DCs previously treated with 2-DG (23). In the currently study, we directly demonstrated defects in both proliferation and inflammatory cytokine production in Glut1^T-KO^ alloreactive T cells without simultaneously targeting non-T cells.

Glut1^T-KO^ T cells previously demonstrated reduced viability compared to WT control after stimulation with plate-bound antibodies (15). To determine whether apoptosis is involved in increased cell death of Glut1^T-KO^ T cells, we assessed the expression of proteins involved in the regulation of apoptotic pathway, including Mdm2, Puma, Noxa, and Mcl-1. Proteins linked to the induction of apoptotic pathway were significantly upregulated as opposed to downregulation of the anti-apoptotic Mcl-1 in activated Glut1^T-KO^ T cells compared to WT control (**Figure 3A**). Specifically, Puma, which is sensitive to rapid upregulation in response to glucose deprivation to promote apoptosis (53), was drastically induced in Glut1^T-KO^ T cells, suggesting that glycolysis plays a critical role in regulating apoptosis in activated T cells. We also assessed Mdm2 expression due to its role in regulating cellular stress response and apoptosis. We demonstrated that Mdm2 is significantly increased expression in Glut1^T-KO^ T cells (**Figure 3A**). We further evaluated whether alloreactive T cells are susceptible to apoptosis due to nutrient availability and cellular stress in the context of glycolysis. Annexin V and 7AAD staining confirmed that activated alloreactive Glut1^T-KO^ T cells are prone to undergo apoptosis (**Figure 3B**). BH3-only Bcl-1 family members, including Bim, has been implicated in lymphocyte cell death during prolonged glucose deprivation (53). We showed that alloreactive Glut1^T-KO^ T cells upregulate Bim expression (**Figure 3C**), an indicator for ER stress and disruption of glucose metabolism (36, 53, 54). Mcl-1, a prosurvival factor, is also linked to glycolysis and metabolic stress (35, 38, 55). Regulated post-translationally, Mcl-1 is rapidly stabilized following TCR crosslinking (56, 57) and couples with Noxa to modulate the apoptosis threshold (38). With a short half-life of 30 min, Mcl-1 has a rapid turnover rate and is highly sensitive to changes in global translation downstream of mTORC1 (58). Indeed, Glut1^T-KO^ T cells are incapable of sustaining mTORC1 activation (**Figure 2C**) and Mcl-1 expression (**Figure 3D**) during alloantigen challenge. It is possible that Mcl-1 expression is regulated by mTORC1 in response to glucose utilization to regulate T cell survival. Overall, the above findings demonstrate increased apoptosis induction in activated Glut1^T-KO^ T cells compared to WT control. Interestingly, a previous study showed that viability was only slightly reduced in T cells following stimulation in the presence of 2-DG (59). While these findings appear to be contradictory to the current study, this is potentially attributed to the difference in 2-DG concentration as a higher concentration was used in the current study. Importantly, the timing of 2-DG addition is different. Whereas 2-DG was added at the beginning of stimulation in the previous study, the current assays involved 2-DG addition 16 hours following stimulation, which preferentially affects already activated T cells that are highly sensitive to glycolysis usage.

Since allo-HSCT is the primary curative option for malignant leukemia and lymphomas, it is critical to assess the impact of glycolysis inhibition on GVL activity. Our results demonstrated for the first time that Glut1^T-KO^ T cells provide superior protection in recipients against tumor growth compared to TCDBM + tumor recipients (**Figures 4C, H**). Although GVHD was not completely eliminated with transfer of Glut1^T-KO^ T cells, both survival and body weight are significantly improved in comparison to WT T cell recipients (**Figures 4A, B, F, G**). The reduced but detectable GVHD development (**Figures 4B** and **S6**) in Glut1^T-KO^ T cell recipients may be contributed by metabolic processes other than glycolysis, including glutaminolysis and pentose phosphate pathway (20), hence simultaneous targeting of the above pathways is likely to further improve the abrogation of GVHD. However, the data support that Glut1^T-KO^ T cell retain the capacity to eliminate tumor development (**Figures 4C, H**). Previous studies suggest that expression of cytotoxic granules, such as granzyme B and perforin in CD8^+^ T cells, are not regulated by aerobic glycolysis (11), potentially mediating GVL effect in Glut1^T-KO^ T cells. Despite the earlier onset of apoptosis in glycolysis-inhibited alloreactive CD8^+^ T cells compared to CD4^+^ T cells (**Figure S9B**), ex vivo stimulated T cells were capable of controlling tumor development and improving survival outcome compared to TCDBM + tumor and untreated donor T cell recipients (**Figure 6**).

Furthermore, Glut1^T-KO^ CD8^+^ T cells, which exhibited higher TNFα expression compared to control T cells (**Figure 2H**), suggesting a potential contribution to tumor killing mediated by Glut1^T-KO^ CD8^+^ T cells. Glut1^T-KO^ T cells may also be able to facilitate GVL without meeting the threshold for GVHD induction, as the T cell dose to induce GVHD appears to be 10-fold higher than GVL in clinical studies (60–63). IFN-γ production (**Figure 2G**) and OXPHOS-dependent memory T cells can also contribute to GVL activity (64–66). Collectively, we showed that T cells with impaired glycolysis retained the capacity to prevent tumor development in allogeneic recipients. Interestingly, studies by Uhl demonstrated that leukemia-derived lactic acid interferes with both glycolysis and OXPHOS in T cells, leading to reduced protection against tumor, which appears to be contradictory to the current finding (67). However, Glut1^T-KO^ T cells demonstrate comparable basal OCR level to WT control (**Figure S2D**), suggesting that the preservation of OXPHOS may be critical for GVL preservation in Glut1^T-KO^ T cell recipients. In regard to the metabolic flexibility of T cells, previous studies showed that glucose deprivation in activated T cells can be partially compensated by increased respiration (59). In addition, a recent study demonstrated that CD8^+^ T cells can utilize inosine as an alternative carbon source when glucose utilization is restricted to mediate tumor-killing in xenograft models (68). Hence it is possible that the metabolic plasticity of CD8^+^ T cells contributes to GVL preservation when glycolysis is impaired.

To evaluate the therapeutic potential in a clinically-relevant setting, we sought to assess selective inhibition of alloreactive T cells to remedy off-target effects on other cell types that utilize glycolysis. In line with this approach, we and others previously examined ex vivo treatments to eliminate T cells activated by recipient antigens (69–71). In the currently study, 2-DG strongly induced apoptosis in activated alloreactive T cells (**Figure 5C**). We further validated this approach using both murine and human models, where alloresponse was subdued upon alloantigen rechallenge while response against nonspecific stimulation remained intact (**Figures 5D, E**). The ex vivo assay using 2-DG also demonstrated the translational value of targeted glycolysis inhibition prior to transplantation (**Figures 6A–D**). As expected, recipients of control T cells treated with media only demonstrated reduced survival with increased incubation time. In contrast, optimal 2-DG inhibition for 48 hours yielded significantly improved survival without losing GVL effect compared to recipients of untreated T cells or those treated with media control. Protection against both tumor and GVHD development through 2-DG inhibition for 48 hours was further evaluated using a second tumor model with the A20 cell line, highlighting the therapeutic potential of ex vivo glycolysis inhibition (**Figures 6E–H**). Overall, we observed that the desired efficacy for acute GVHD suppression and GVL can be achieved through selective inhibition of alloreactive T cells ex vivo (**Figure 6**).

In summary, by using T cells genetically incapable of utilizing glycolysis, we demonstrated that glycolysis is definitively required for alloreactive T cells to induce acute GVHD. We further established a role for glycolysis in promoting donor T cell pathogenicity through regulating proliferation, cell death, and proinflammatory cytokine production. One potential limitation of 2-DG glycolysis inhibition in the clinical setting is its application as a preventative procedure but not as a curative treatment due to toxicity if administered systemically. However, reagents better tolerated for allogeneic BMT with low toxicity can be used as a curative treatment, including 3-(3-pyridinyl)-1-(4-pyridinyl)-2-propen-1-one, which has been assessed in a murine GVHD model (20). Overall, the current study demonstrated that we can target glycolysis in alloreactive T cells to prevent acute GVHD without losing the GVL activity.

## Data Availability Statement

The original contributions presented in the study are included in the article/**Supplementary Material**. Further inquiries can be directed to the corresponding author.

## Ethics Statement

The animal study was reviewed and approved by Institutional Animal Care and Use Committee (IACUC) of the Duke University Medical Center. Written informed consent was obtained from the owners for the participation of their animals in this study.

## Author Contributions

YH, YZ, and BJC designed the research studies, analyzed the data. YH, YZ, YJ, PS, XN, WH, CX, and AN participated in conducting experiments and acquiring data. YZ and BJC participated in writing the manuscript. YH, YZ, BJC, ANM, NJM, DMC, TVB, ZL, NJC, MC, CH participated in editing the manuscript. YZ, BJC, and NJC participated in revising the manuscript. DMC performed pathological analysis. FL and CL generated A20 cells expressing Luc-EGFP gene. All authors contributed to the article and approved the submitted version.

## Funding

This work was supported by grant P01 CA047741.

## Conflict of Interest

The authors declare that the research was conducted in the absence of any commercial or financial relationships that could be construed as a potential conflict of interest.

## Publisher’s Note

All claims expressed in this article are solely those of the authors and do not necessarily represent those of their affiliated organizations, or those of the publisher, the editors and the reviewers. Any product that may be evaluated in this article, or claim that may be made by its manufacturer, is not guaranteed or endorsed by the publisher.
