# Supplementary material for: Targeting Glycolysis in Alloreactive T Cells to Prevent Acute Graft-Versus-Host Disease While Preserving Graft-Versus-Leukemia Effect
Source: Front Immunol. 2022 Feb 28;13:751296. doi: 10.3389/fimmu.2022.751296 (PMC8920494; doi:10.3389/fimmu.2022.751296)
Supplement: Supplementary file 1 [file DataSheet_1.pdf]

Supplemental Material

Targeting glycolysis in alloreactive T cells to prevent acute graft-versus-host disease while preserving graft-versus-leukemia effect

Ying Huang, Yujing Zou, Yiqun Jiao, Peijie Shi, Xiaoli Nie, Wei Huang, Chuangfeng Xiong, Michael Choi, Charles Huang, Andrew N. Macintyre, Amanda Nichols, Fang Li, Chuan-Yuan Li, Nancie J. MacIver, Diana M. Cardona, Todd V. Brennan, Zhiguo Li, Nelson J. Chao, Jeffrey C. Rathmell, and Benny J. Chen

Supplemental Figures

A

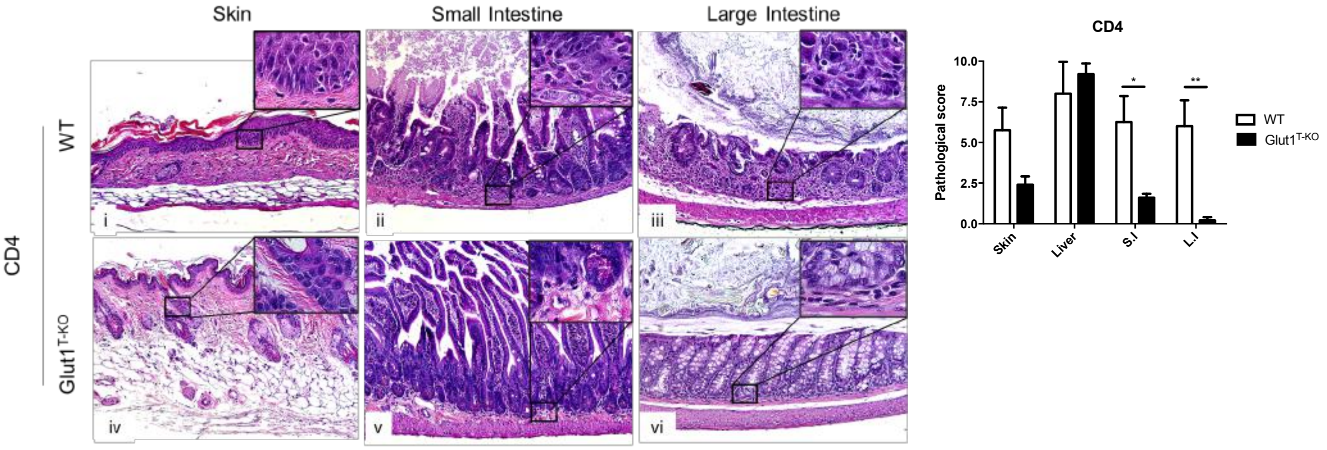

B

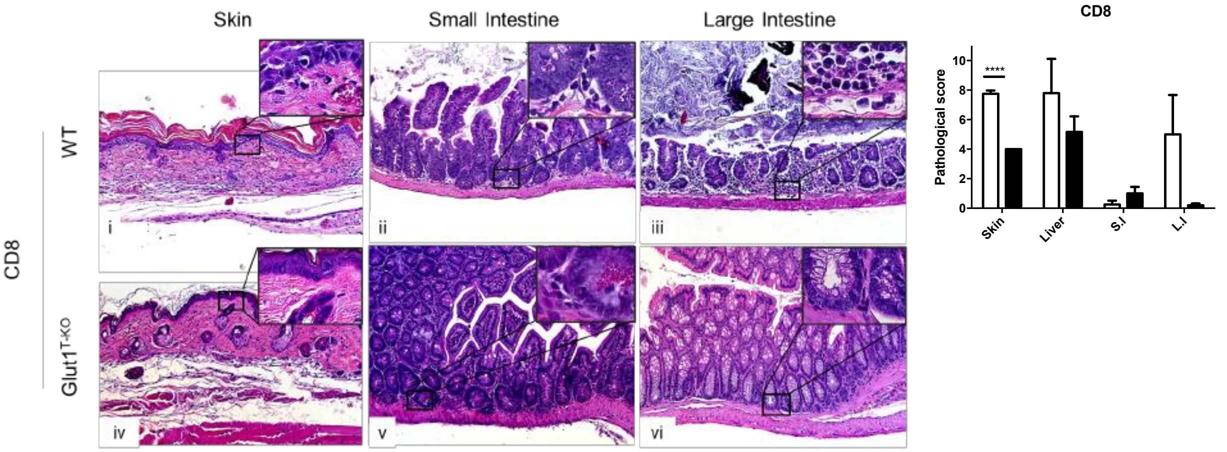

Figure S1. Transfer of Glut1$^{T\text{-}KO}$ T cells alleviates recipient tissue damage. Acute GVHD was induced by transplantation of C57BL/6-derived 1 x 10^7 TCDBM or together with 1 x 10^6 WT or Glut1$^{T\text{-}KO}$ T cells into lethally irradiated (8.5 Gy) BALB/c recipients. Tissues were collected for H&E histology (10x & 40x) (A) of skin (i), small intestine (ii), and large intestine (iii) in recipients for WT CD4 T cells; skin (iv), small intestine (v), and large intestine (vi) in recipients for Glut1$^{T\text{-}KO}$ CD4 T cells. (B) H&E histology of skin (i), small intestine (ii), and large intestine (iii) in recipients for WT CD8 T cells; skin (iv), small intestine (v), and large intestine (vi) in recipients for Glut1$^{T\text{-}KO}$ CD8 T cells. Pathological scores were determined for recipient specimens. Data are shown as mean ± SEM, *$P < 0.05$, **$P < 0.01$, ****$P < 0.0001$ (Glut1$^{T\text{-}KO}$ vs. WT); 2-tailed Student t test. Data are representative of three independent experiments. S.I., small intestine; L.I., large intestine.

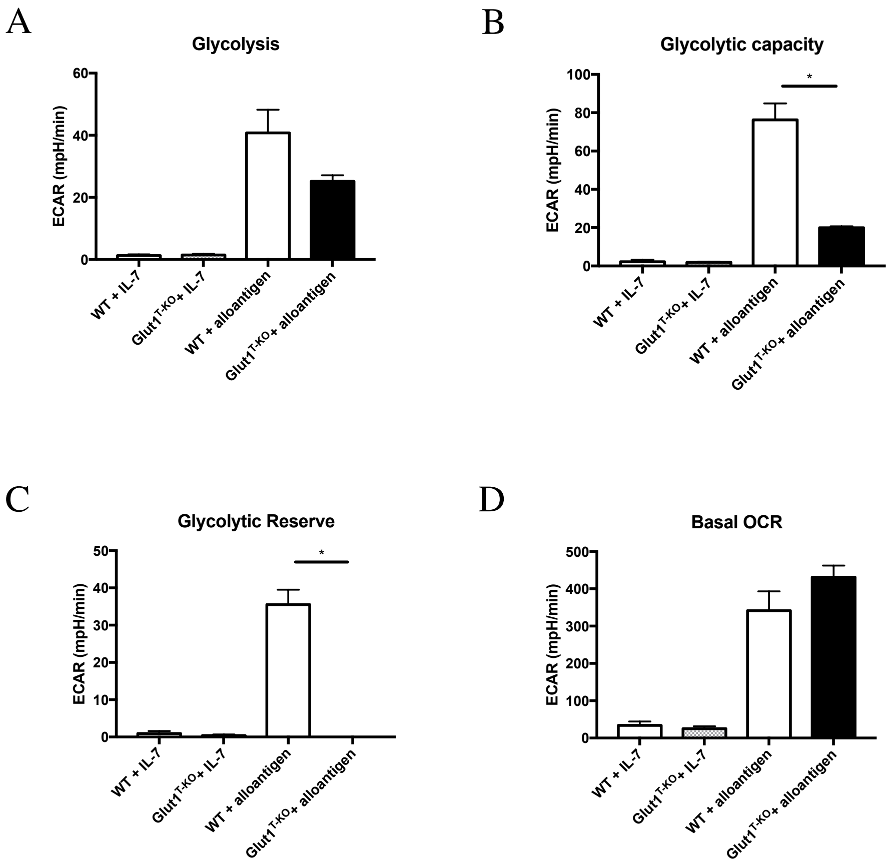

Figure S2. Glut1 is required for the metabolic reprogramming of activated alloreactive T cells. WT or Glut1$^{T\text{-}KO}$ T cells were stimulated using irradiated (20 Gy) BALB/c splenocytes or cultured with IL-7. (A) glycolysis, (B) glycolytic capacity, (C) glycolytic reserve, and (D) Basal oxygen consumption rate (OCR) were measured after 5 days. Data are representative of two or more experiments (n = 3) and are shown as mean ± SEM. *P < 0.05, 2-tailed Student t test.

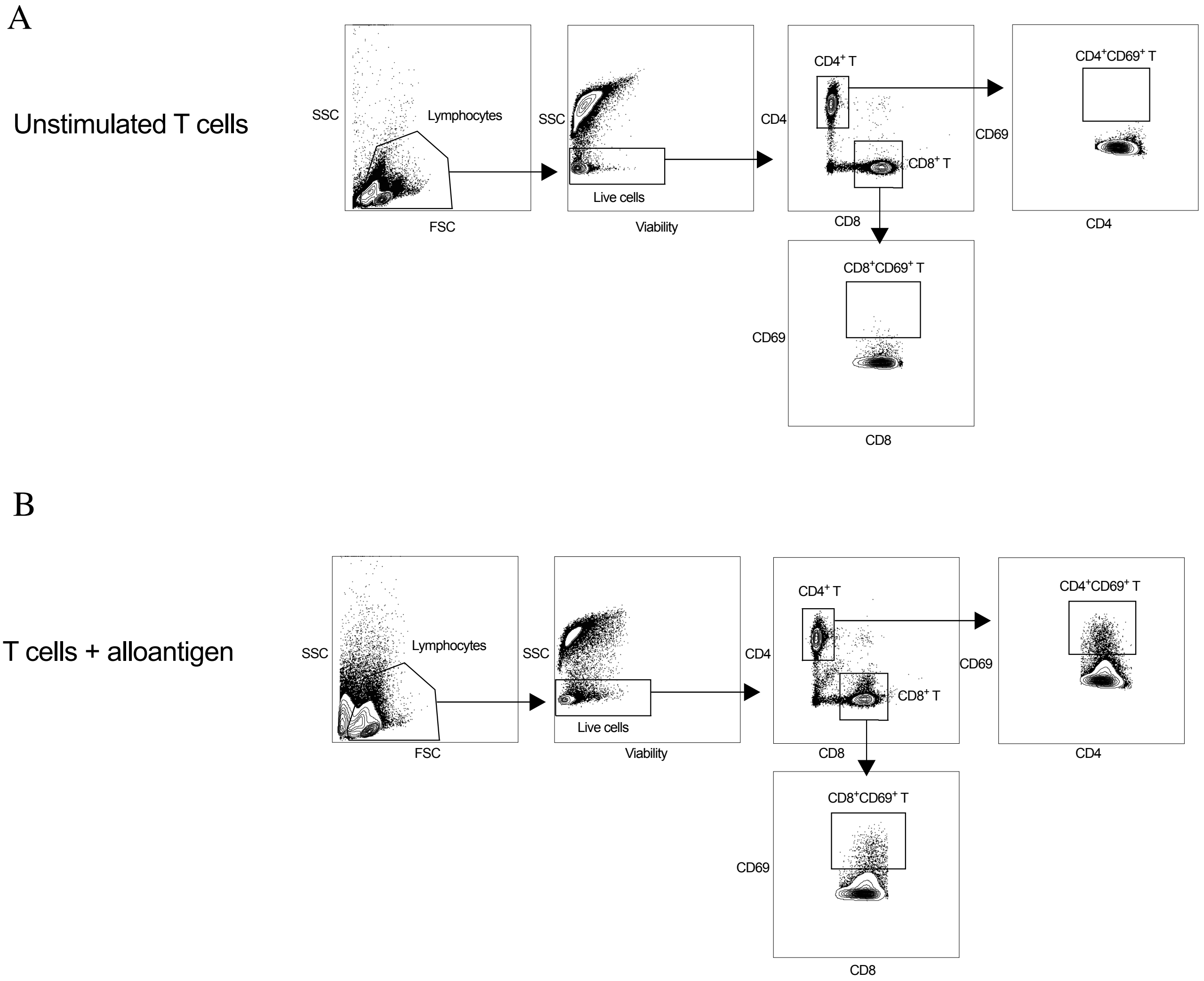

Figure S3. Flow cytometry gating strategy for alloreactive T cells. WT or Glut1$^{T\text{-}KO}$ T cells were untreated or stimulated using irradiated (20 Gy) BALB/c dendritic cells, then stained for T cell markers CD3, CD4, CD8, activation marker CD69, and fixable viability dye. (A) Gating strategy for identifying unstimulated T cells. Live lymphocytes were defined by low forward and side scatter (FSC and SSC) followed by negative staining for the cell death marker. CD4 or CD8 T cells negative for CD69 were further gated from live cells. (B) Gating strategy for identifying stimulated T cells. Gates for activated CD4 or CD8 T cells were defined using untreated T cells as a negative control.

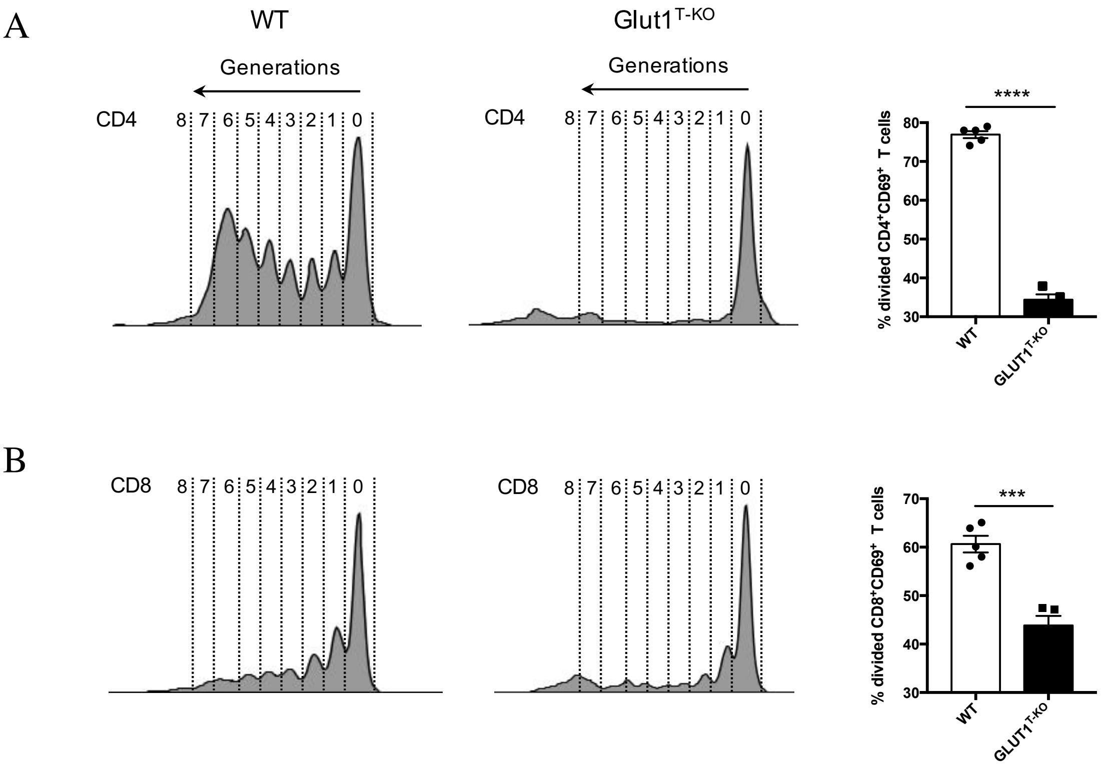

Figure S4. Glut1 mediates the expansion of activated alloreactive T cells. Following transfer of CTV-labelled WT or Glut1$^{T\text{-}KO}$ T cells, along with B6.SJL TCDBM into lethally irradiated BALB/c recipients, T cell proliferation was analyzed (left panel) and measured by frequency (right panel) 58 hr later. Data are representative of three experiments (n = 3) and are shown as mean ± SEM. ***$P < 0.001$, ****$P < 0.0001$, 2-tailed Student t test.

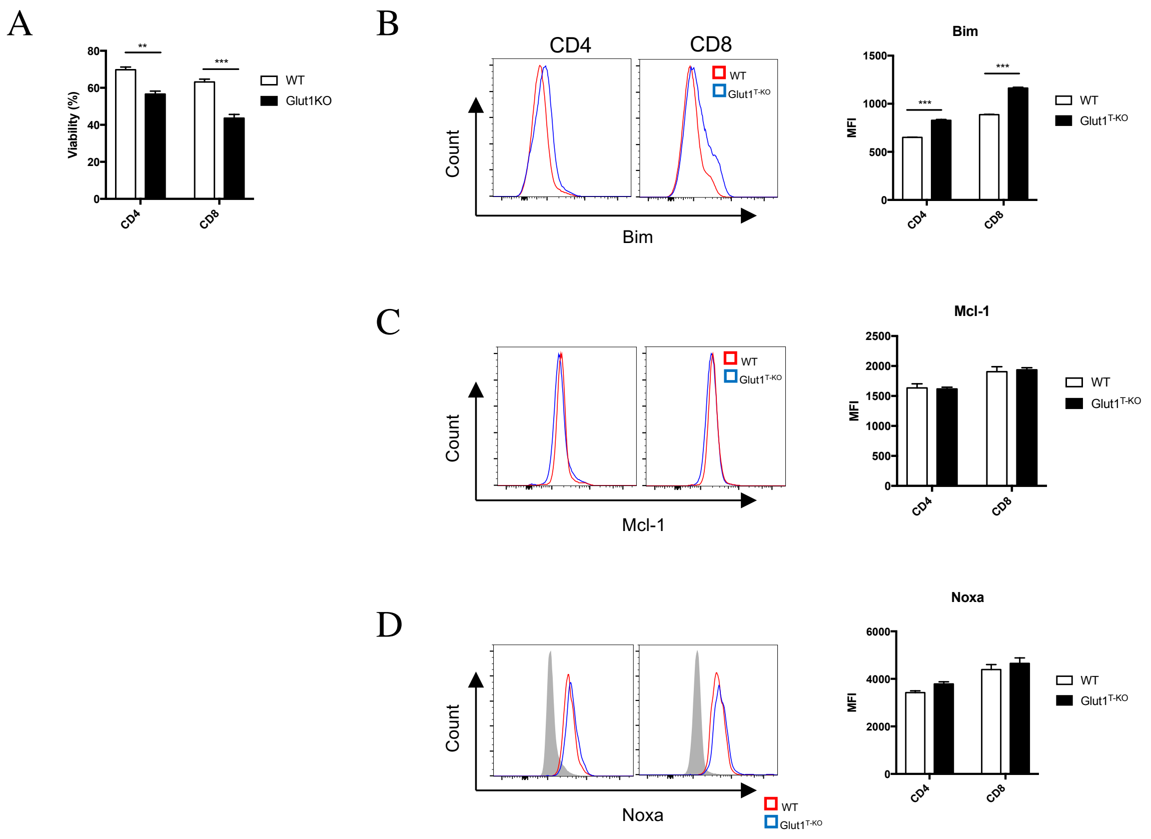

Figure S5. Expression of proteins in the apoptotic and anti-apoptotic pathways in resting and alloreactive WT and Glut1$^{T\text{-}KO}$ T cells. WT or Glut1$^{T\text{-}KO}$ T cells were stimulated for 16 hours by irradiated (20 Gy) BALB/c DCs and analyzed by flow cytometry for staining of the viability marker (A). Resting WT or Glut1$^{T\text{-}KO}$ T cells were assessed for Bim (B) and Mcl-1 (C) following 16 hours of culture. Following 16-hour incubation with irradiated BALB/c DCs, alloreactive WT or Glut1$^{T\text{-}KO}$ T cells were stained for Noxa expression (D). Data are representative of two or more experiments (n = 3) and are shown as mean ± SEM. $^{**}P < 0.01$, $^{***}P < 0.001$, 2-tailed Student t test.

A

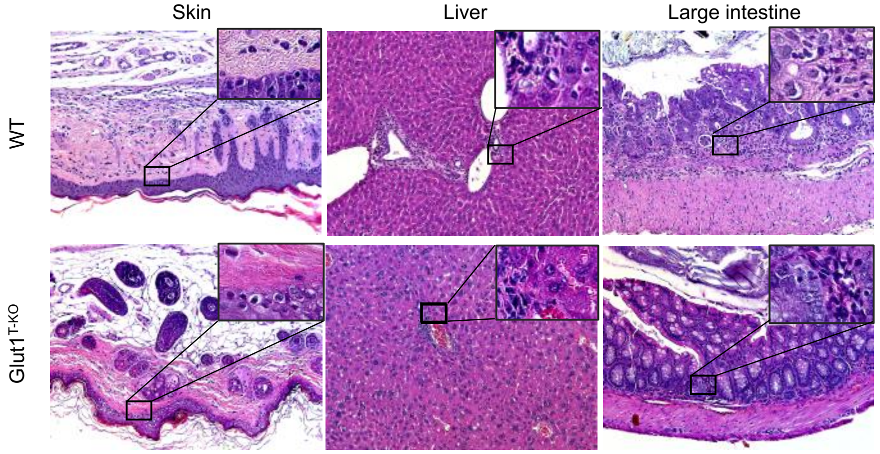

B

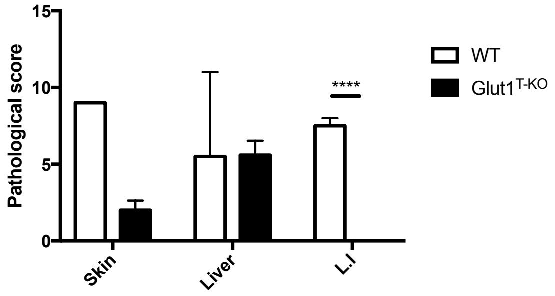

Figure S6. Transfer of Glut1$^{T\text{-}KO}$ T cells reduces but does not completely eliminate damage to target organs. Lethally irradiated (8.5 Gy) BALB/c recipients were transplanted with 1 x 10^6 WT or Glut1$^{T\text{-}KO}$ T cells, along with 1 x 10^7 TCDBM and 5 x 10^5 host-type BCL1 cells. H&E histology (10x & 40x) (A) and pathological scores of target organs (B) from recipients was assessed (time at sample collection: TCDBM alone, day 21; WT, day 27; Glut1$^{T\text{-}KO}$, day 105).

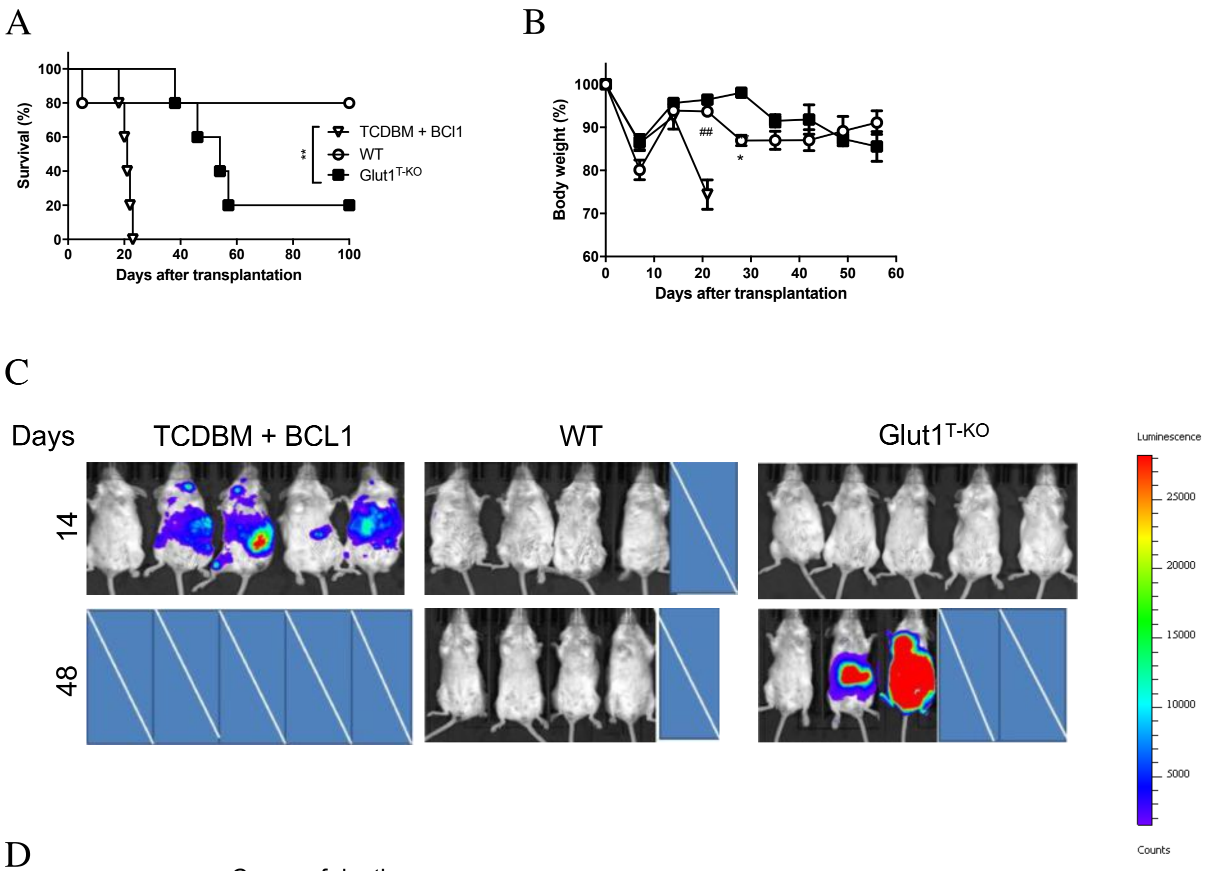

D

Cause of death

	GVHD	Tumor
TCDBM + BCL1	0/5	5/5
WT	1/5	0/5
Glut1^{T-KO}	0/5	4/5

Figure S7. Transfer of low dose T cells into recipients with leukemia. Lethally irradiated (8.5 Gy) BALB/c recipients were transplanted with 1 x 10^5 WT or Glut1^{T-KO} T cells, along with 1 x 10^7 TCDBM and 5 x 10^5 host-type BCL1 cells. Recipients were monitored for survival (A) and body weight (B) up to 100 days after transplantation. Development of leukemia/lymphoma (C) was monitored by BLI. Cross symbols indicate death prior to imaging collection. Cause of death due to GVHD or tumor development was summarized (D). **P < 0.01, **P < 0.001, log-rank test (A); *P < 0.05, **P < 0.01 (Glut1^{T-KO} vs. WT); $^{\#\#}$P < 0.01 (TCDBM vs. WT), 2-tailed Student t test (B). data are representative of three experiments (n = 5).

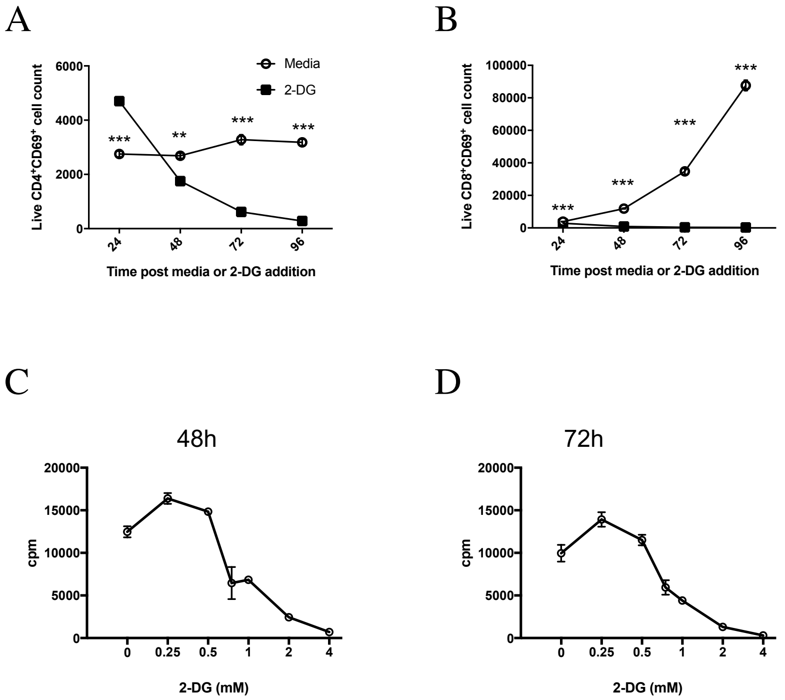

Figure S8. 2-DG treatment of activated T cells inhibits alloresponse. C57BL/6 T cells were stimulated with BALB/c dendritic cells for 16 hr, followed by the addition of 2-DG or media control for indicated periods. Number of live $CD4^+CD69^+$ (A) and $CD8^+CD69^+$ cells (B) were assessed by the end of the culture. 4C T cells were stimulated with BALB/c splenocytes for 16 hr, followed by treatment with media control or 2-DG for 48h (C) or 72h (D). Cells were collected for thymidine incorporation assay at 112h. Data are representative of two experiments (n = 3) and are shown as mean ± SEM.

A

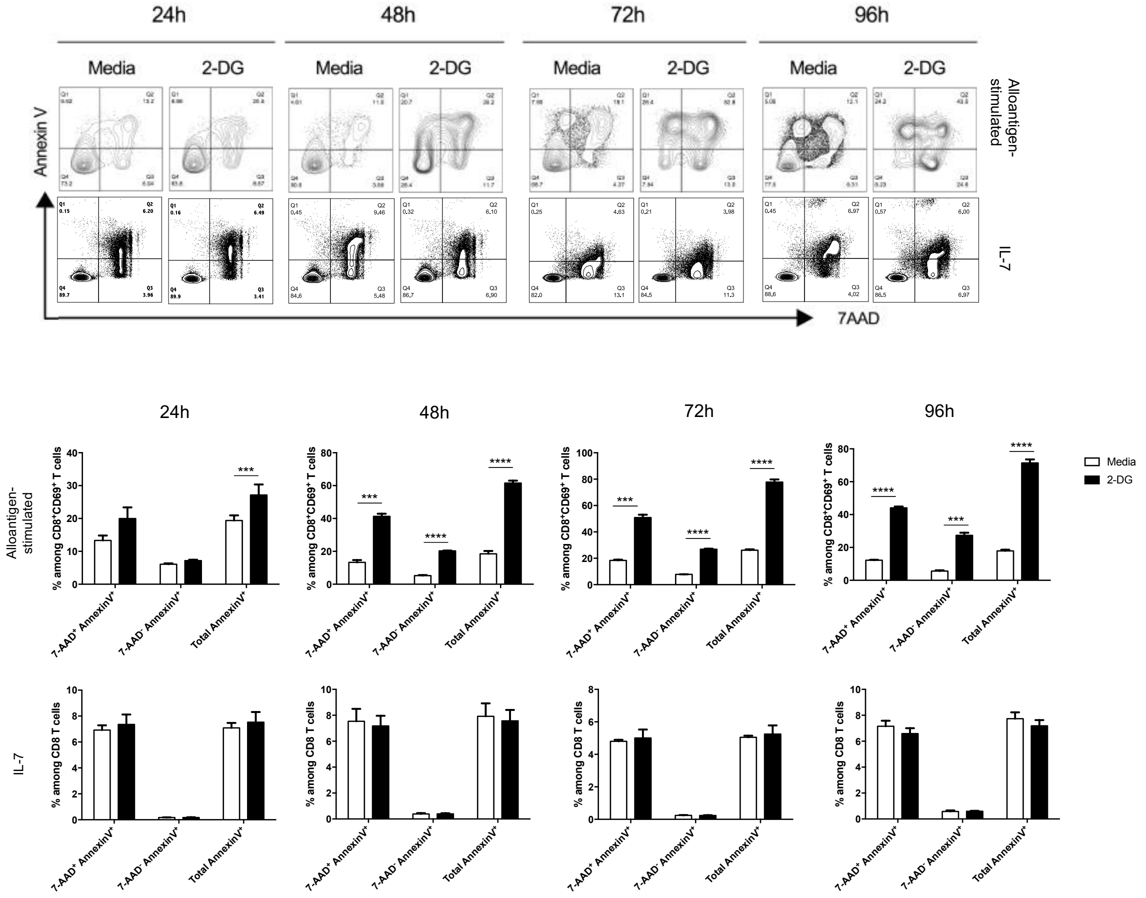

B

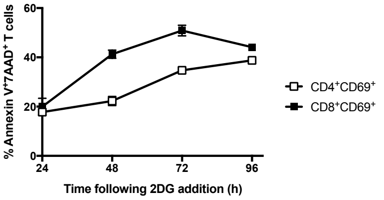

Figure S9. 2-DG treatment selectively suppresses alloreactive CD8^{+}T cells but not IL-7-treated CD8^{+}T cells. WT CD8^{+} T cells were first stimulated with irradiated (20 Gy) BALB/c DCs for 16 hours, followed by incubation with freshly isolated irradiated DCs in the presence of media control or 8mM 2-DG for indicated time points; WT CD8^{+} T cells were cultured in IL-7 (10ng/ml) for 16 hours plus indicated culture times and analyzed for Annexin V and 7AAD (A). Upper panel, representative flow cytometry plots; lower panel, quantification for percentage of 7AAD^{+}Annexin V^{+}, 7AAD^{-}Annexin V^{+}, and total Annexin V^{+} CD8^{+} T cells. T cells were gated on CD8^{+} CD69^{+} for alloantigen-stimulated samples and CD8^{+} for IL-7-treated samples. (B) percentage of CD4^{+} CD69^{+} and CD8^{+} CD69^{+} T cells that are 7AAD^{+}Annexin V^{+} in 2-DG-treated T cells stimulated with BALB/c DCs.

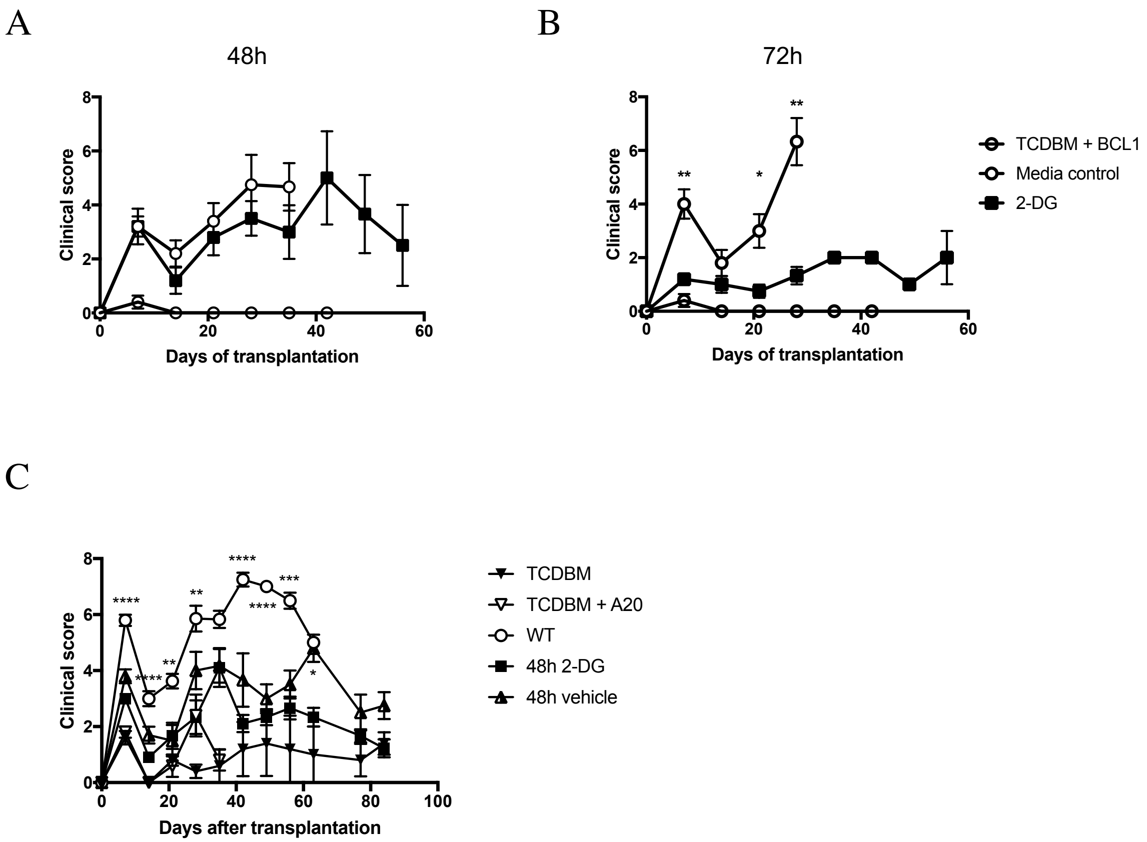

Figure S10. 2-DG inhibition of alloreactive donor T cells alleviates GVHD development in different GVL models. T cells were stimulated with BALB/c dendritic cells for 16 hr, followed by the addition of 8mM 2-DG for indicated periods were transplanted into BALB/c recipients, along with 1×10^7 TCDBM and 1×10^5 BCL1 cells. Clinical scores were recorded for recipients of T cells treated with 2-DG for 48 hr (A) and 72 hr (B). In the second GVL model, T cells were stimulated with BALB/c dendritic cells for 16 hr then treated with 8mM 2-DG for 48 hr were transplanted into BALB/c recipients, along with 1 x 10^7 TCDBM and 1 x 10^5 A20 cells. Clinical scores were recorded (C). Data are representative of two experiments (n = 5, (A) and (B); n = 10, (C)) and are shown as mean ± SEM. *P < 0.05, **P < 0.01, ***< 0.001, ****< 0.0001. 2-tailed Student t test.
